# Nano-priming modulates antioxidant enzymes and NHX/SOS-mediated ion homeostasis to improve salinity tolerance in barley genotypes

**DOI:** 10.1007/s00299-026-03798-6

**Published:** 2026-04-13

**Authors:** Wesam W. Abozaid, Samar G. Thabet, Mohamed A. Karam, Yasser S. Moursi

**Affiliations:** Department of Botany, Faculty of Science, University of Fayoum, Fayoum, 63514 Egypt

**Keywords:** Barley, Salinity stress, Nano-priming, Gene expression, *CAT1*, *NHX*

## Abstract

**Key message:**

By controlling biochemical function and stress-related gene expression, ZnO nano-priming improved barley salinity tolerance beyond hydro-priming, demonstrating its coordinated biochemical and molecular effects.

**Abstract:**

Salinity stress is a significant abiotic constraint that disrupts redox homeostasis and ionic equilibrium of barley. Nano-priming has emerged as an effective tool to mitigate these impacts by improving stress-responsive pathways. To investigate the biochemical and molecular responses to salt stress, 10 contrasting genotypes were selected from 170 genotypes screened under control conditions and at 200 mM NaCl using unprimed, hydro-primed, and nano-primed seeds. Biochemical traits, including protein, carbohydrates, proline, mineral absorption, and antioxidant activity, were assessed. QPCR was used to quantify the expression of 10 stress-related genes (CAT1, APX, SODA, GPX, GR, NHX1, NHX2, NHX3, SOS1, SOS3). Compared to unprimed and hydro-primed seeds, nano-priming significantly increased osmolyte accumulation, mineral absorption, and antioxidant enzyme activity. Principal component analysis and hierarchical clustering confirmed significant genotype-specific responses, with ionic traits (K⁺, K⁺/Na⁺) and antioxidant enzymes (CAT, GST, POD) driving separation under salinity. Under salinity, CAT1 was strongly expressed in the tolerant genotype HOR11370 (79.02-fold), while NHX3 was highly expressed in the tolerant genotype BCC1398 (208.16-fold). Nano-priming also significantly upregulates CAT1 in the sensitive genotype BCC532. These findings provide molecular evidence that nano-priming enhances barley resilience by reprogramming gene expression, ion transport, and antioxidant pathways. The differential regulations within and among tolerant and sensitive genotypes indicate that the integration of multiple mechanisms confers salinity tolerance in barley. These findings provide valuable insights into breeding strategies to improve crop performance under saline conditions.

**Supplementary Information:**

The online version contains supplementary material available at 10.1007/s00299-026-03798-6.

## Introduction

A worldwide concern, soil salinization impacts 1100 Mha of soil, or around 7% of the planet's land area (Balasubramaniam et al. 2023; Sahbeni et al. 2023). Salinity induces severe morpho-physio-biochemical disruptions in plants because of metabolic alterations, nutritional imbalances, and cell membrane damage (Alsamadany et al. 2023). Salinity primarily results in osmotic changes in the rhizosphere, reducing plant roots' water capacity and nutrient uptake, leading to ionic toxicity. The excessive buildup of Na^+^ and Cl^–^ restricts the absorption of nutrients, resulting in K^+^ deficiency, high Na^+^/K^+^ ratio, and enzyme inactivation (Taha et al. 2020). Salt tolerance is a complex trait, governed by multiple genes and integrated physiological and biochemical processes (Zhang and Shi 2013). Plants can adapt to the saline environments through morphological, physiological, biochemical, and molecular changes, including ion homeostasis and compartmentalization, osmotic adaptation, and enhanced antioxidation metabolism, to scavenge ROS (Arif et al. 2020; Balasubramaniam et al. 2023; Zhang and Shi 2013). Proline, an essential compatible osmolyte and antioxidant, accumulates under salinity stress and helps plants keep cell turgor. The increased proline content has been recognized and used as a physiological indicator of the plant's response to salinity stress (Abdelhamid et al. 2013; Balasubramaniam et al. 2023). Salinity affects seed germination by disrupting water imbibition, altering enzymatic activities that result in ionic toxicity, interfering with protein metabolism, causing hormonal imbalances, and decreasing the likelihood that seeds will use their reserves, delaying and reducing the number of seeds that sprout (Debez et al. 2020; Munns et al. 2012).

Barley, the fourth most significant crop globally (Schulte et al. 2009; Visioni et al. 2019), is classified as a glycophyte; yet its resistance to salinity differs among genotypes (Flowers and Hajibagheri 2001; Xue et al. 2009). It is an appropriate model cereal crop for studying the genetics of developmental and adaptation features because of its significant genetic variation regarding stress tolerance (Dawson et al. 2015). Certain barley genotypes can flourish in saline environments (Shen et al. 2018). Salinity-tolerant barley genotypes demonstrate halophytic characteristics, including the exclusion of Na^+^ from absorption (Chen et al. 2007) and the accumulation of Na^+^ in tissues (Munns and Tester 2008). Tolerant genotypes trap Na^+^ in intracellular vacuoles, thereby preserving elevated K^+^/Na^+^ ratios in the cytosol and mitigating Na^+^ toxicity (Han et al. 2018; Mian et al. 2011). To balance the osmotic potential of vacuolar Na^+^, they can also create suitable solutes in the cytoplasm (Widodo et al. 2009).

A variety of genes that exhibit differential expression during plant development regulate salinity tolerance (Qiu et al. 2011). The Na^+^/H^+^ antiporter (*NHX*) is a universal intercellular transporter present in all living organisms. According to Bassil et al. (2019), these genes are necessary for osmotic modulation, stomatal regulation, and floral formation. The Arabidopsis thaliana *NHX* isoforms (*AtNHX1-4*) have significant vacuolar localization, and the other class of *NHX* proteins (*AtNHX5-6*) show endosomal localization in cells (Barragán et al. 2012). In barley, four *NHX* isoforms are mainly located in the vacuole: *HvNHX1*,* HvNHX2*,* HvNHX3*, and *HvNHX4* (Jabeen et al. 2022). In general, the expression of these genes during salt stress is species-specific and varies according to the degree of salinity sensitivity (Jabeen et al. 2022). The Salt-Overly-Sensitive (SOS) signaling transduction pathway is one of the principal regulatory systems for preserving ion homeostasis and protecting different tissues and organs from excessive and persistent salt (Ji et al. 2013). This process involves three genes, *SOS1*, *SOS2*, and *SOS3*, whose expression is triggered by salinity stress (Shi et al. 2002). As the salinity increases in the rhizosphere, Na^+^ enters root tissues, including cortex, endodermis, and xylem tissues. The *SOS1* gene controls the compartmentalization of Na^+^ and its loading to the xylem in this situation. It was previously demonstrated that *SOS1* is essential for the loading of Na^+^ in the xylem (Olías et al. 2009). Barley's excellent tolerance to salinity stress makes it an ideal model crop for research on stress adaptation. Thus, analysis of biochemical and molecular mechanisms can identify key genes involved in barley's ability to withstand salt and shed light on trade-offs among phenotypic and transcriptomic traits. While these pathways have been studied individually, integrated analyses linking biochemical traits with gene expression remain limited in barley.

Seed priming has emerged as a practical approach to enhance stress tolerance by preconditioning seeds to adverse environments. Conventional methods such as hydro-priming have shown moderate success; however, recent advances highlight the potential of nano-priming. Among various nanomaterials tested for alleviating abiotic stress, the use of zinc oxide nanoparticles (ZnO NPs) is considered one of the most promising strategies for reducing abiotic stress in plants (Alhammad et al. 2023). Zinc (Zn) is an essential micronutrient that functions as a cofactor for multiple regulatory enzymes, playing a role in photosynthesis, the biosynthesis of various metabolites, and the cellular homeostatic response (Zlobin 2021). Synthetic zinc fertilizers are thought to be less effective sources of zinc when used in agriculture (Fatima et al. 2021). As a result, Zn-NPs can be a useful tool for reducing the serious issues caused by salinity in agriculture (Seleiman et al. 2020). Previous studies have reported that seed priming with zinc oxide nanoparticles has been shown to mitigate both abiotic and biotic stresses in plants. Acting as a biostimulant, ZnO NPs enhance germination rates, seedling establishment, nutrient uptake, fresh weight accumulation, biomass production, and photosynthetic efficiency (Donia and Carbone 2023). Under salinity stress, treating ZnO-NPs can improve the plant’s morphological, physiological, and biochemical characteristics (Zulfiqar and Ashraf 2021). ZnO NPs, in particular, have shown efficacy across multiple species, including wheat, barley, rapeseed, tomato, and rice (Adil et al. 2022; Ali et al. 2022; El-Badri et al. 2021; Faizan et al. 2021Singh et al. 2022). However, these effects are often concentration-dependent, with optimal doses improving growth and antioxidant capacity, whereas higher concentrations may induce inhibitory responses as demonstrated by Srivastav et al. (2021), who examined the growth and biochemical responses of maize and wheat to a range of ZnO NPs (0–200 mg L^−1^). According to their findings, the treatment of 100 mg ZnO NPs L^−1^ considerably enhanced growth and antioxidant enzyme activity, whereas the higher concentrations (i.e., 150–200 mg) caused variable reductions.

Despite these promising outcomes, the molecular basis of nano-priming in barley remains poorly understood, and genotype-specific responses have not been systematically investigated. Without a clear understanding of how nano-priming influences both biochemical and transcriptional pathways across tolerant and sensitive genotypes, its integration into breeding and agronomic strategies remains limited. Therefore, in this study, we specifically investigated the role of ZnO NPs in mitigating salt stress, aiming to provide new insights into their potential application in crop improvement. The goals of the current study were to 1) analyze how zinc oxide nanoparticles and hydro-priming affect the biochemical responses, and ion homeostasis in relation to salinity tolerance during early development in contrasting barley genotypes and 2) examine the effect of zinc oxide nanoparticles on the expression patterns of salinity tolerance-related genes in tolerant and sensitive genotypes to better understand the molecular basis of salinity tolerance.

## Materials and methods

### Plant materials

The eleven genotypes included in the current study were selected among a large set of 170 diverse genotypes that were tested under salinity stress (200 mM NaCl) during seed germination and seed set under different seed treatments (unprimed, hydro-primed, and nano-primed). Detailed information for each accession, including its biological status (landrace, modern cultivar, or wild barley) and geographical origin, is presented in Table 1. The seeds were obtained from the IPK Gene Bank, Gatersleben, Germany.

**Table 1:** Details about the classification of eleven contrasting barley genotypes (six tolerant and five sensitive genotypes), accession name, biological status, region of origin, country of origin, and row number

BCC Number	Type	Accession name	Name	Biological status^#^	Region of origin	Country of origin	Row number	Biochemical traits	Gene expression
BCC1398	Tolerant	MK 42	Hordeum vulgare L. convar. distichon (L.) Alef. var. nutans (Rode) Alef	breeding/research material	EU	HUN	2	**✓**	**✓**
BCC1416	Tolerant	Tellus	Hordeum vulgare L. convar. distichon (L.) Alef. var. nutans (Rode) Alef	advanced/improved cultivar	EU	SWE	2	**✓**	**✓**
BCC1469	Tolerant	Granal	Hordeum vulgare L. convar. distichon (L.) Alef. var. inerme Körn	advanced/improved cultivar	WANA	KAZ	2	**✓**	**x**
BCC502	Tolerant	Itu Native	Hordeum vulgare L. convar. vulgare var. hybernum Vib	advanced/improved cultivar	EA	CHN	6	**✓**	**x**
BCC538	Tolerant	Rewari	Hordeum vulgare L. convar. vulgare var. hybernum Vib	advanced/improved cultivar	EA	IND	6	**✓**	**x**
HOR11370	Tolerant	MR 1/13	Hordeum vulgare L. convar. distichon (L.) Alef. var. nutans (Rode) Alef	breeding/research material	WANA	ISR	2	**x**	**✓**
BCC1498	Sensitive	K 17227	Hordeum vulgare L. convar. vulgare var. hybernum Vib	traditional cultivar/landrace	WANA	UZB	6	**✓**	**x**
BCC1505	Sensitive	Pallidum 4	Hordeum vulgare L. convar. vulgare var. hybernum Vib	advanced/improved cultivar	EU	UKR	6	**✓**	**✓**
BCC173	Sensitive	IG 128104	Hordeum vulgare L	traditional cultivar/landrace	WANA	PAK	6	**✓**	**x**
BCC526	Sensitive	C-138	Hordeum vulgare L. convar. vulgare var. rikotense Regel	breeding/research material	EA	IND	6	**✓**	**x**
BCC532	Sensitive	Ratna	Hordeum vulgare L. convar. vulgare var. hybernum Vib	advanced/improved cultivar	EA	IND	6	**✓**	**✓**

Where, BCC, Barley Core Collection number; HOR, Hordeum vulgare accession number; EU, Europe; EA  East Asia; WANA,  West Asia and North Africa; HUN, Hungary; SWE, Sweden; KAZ, Kazakhstan; CHN,  China; IND, India; ISR, Israel; UZB, Uzbekistan; UKR, Ukraine; PAK, Pakistan;

**✓**, included genotypes and **x** , excluded genotypes

### Experimental design

The experiment was conducted as previously described in our previous study (Abozaid et al. 2025). Briefly, 170 very different barley genotypes were examined during seed germination and seedling establishment using a full randomized block design with three biological replicates. The experiment consisted of six treatments: control (0 mM NaCl) and salinity stress (200 mM NaCl) under three conditions (unprimed, hydro-priming, and nano-priming (100 ppm of zinc oxide nanoparticles). Seeds were soaked either in distilled water (hydro-priming) or in 100 ppm of zinc oxide (ZnO) nanoparticles (nano-priming) before germination for 12 h in darkness at 10 °C, following the procedure described in our previous work (Abozaid et al. 2025). The ZnO nanoparticles were obtained from Sigma Aldrich. The manufacturer specifies that these ZnO nanoparticles are high-purity, with a nominal primary particle size of 20–30 nm, indicating a nanoscale distribution confined to this range. Following priming, seeds were dried to their initial moisture content, stored in polyethylene bags, and maintained at 4 °C until use. In each treatment category (unprimed, hydro-primed, nano-primed), 20 seeds per genotype were germinated on two filter papers within 9 cm sterile Petri dishes. After priming, 10 mL of distilled water for the control or a 200 mM NaCl solution for salt stress was applied. Petri dishes were maintained at 20 °C in darkness, and germination was defined by radicle emergence of at least 2 mm. Germination and seedling-related traits were assessed for up to 10 days. Genome-wide association analysis (GWAS) was performed using the collected data. Eleven contrasting genotypes (six salinity-tolerant and five salinity-sensitive) were selected based on their performance under salinity. The best six tolerant genotypes were detected using the iPASTIC toolkit, average of sum ranks (ASR) for each trait, and determined by arranging all genotypes from high to low values for all traits. Based on the origin of the selected genotypes, they could be classified into two genotypes from East Asia, two from Europe, and two from West Asia and North Africa. Likewise, the five susceptible genotypes were determined using the above-mentioned approach. Based on the origin of the selected genotypes, they could be classified into two genotypes from East Asia, one from Europe, and two from West Asia and North Africa (Table 1). Ten of those eleven genotypes were chosen for biochemical tests. Five genotypes (three tolerant and two sensitive) were employed for gene expression (Table 1). For each selected genotype under each treatment, three biological replicates were prepared, each consisting of one Petri dish containing 20 seeds. The experiment lasted for ten days, after which the seedlings were collected for analysis. Samples were taken from the whole seedlings.

### Biochemical Assay

For each genotype, seedling tissue samples (three biological replicates) were promptly frozen in liquid nitrogen and preserved at − 80 °C until subsequent analyses. A cooled glass Teflon tissue homogenizer (ST-2 Mechanic-Preczyina, Poland) was used to homogenize plant samples for biochemical analysis. Following homogenization, the supernatants were stored at -20°C in a deep freezer until they were needed for biochemical tests. All samples were later analyzed using a UV–Vis double-beam spectrophotometer (Model T80, PG Instruments Limited, Leicester, United Kingdom). The absorbance was measured at specific wavelengths according to each measured trait.

### Protein determination

Bradford's technique was used to determine the total proteins (Bradford 1976). 100 mg of Coomassie Brilliant blue G-250 was dissolved in 50 mL of 95% ethanol to create the protein reagent.100 mL of 85% (W/V) phosphoric acid was added to this mixture. A final volume of 1 L was achieved by diluting the resultant solution. 50 µL of the sample solution or 50µLof serial concentrations containing 10-100µg bovine serum albumin were pipetted into test tubes to prepare the standard curve. Phosphate buffer (0.1 M, pH 6.6) was used to reduce the test tube's capacity to 1 mL. After adding 5 mL of protein reagent to the test tube, the contents were combined by vortexing or inversion. After 2 min and before an hour, the absorbance at 595 nm was measured against a blank made from 5 mL of protein reagent and 1 mL of phosphate buffer.

### Carbohydrate determination

The total amount of carbohydrates was determined using the phenol–sulfuric acid reaction (Dubois et al. 1956). A boiling tube was filled with around 100 mg of the plant sample. After adding 10 mL of 2.5 N HCl, the mixture was allowed to hydrolyze for 3 h in a boiling water bath, then cooled to room temperature. Sodium carbonate was used to neutralize the solution and produce effervescence. After centrifuging the material, the supernatant was gathered for examination (Sadasivam and Manickam 1992). 100 µL of the extract and 0.5 mL of 20% (w/v) phenol were combined in a colorimetric tube. Five milliliters of strong sulfuric acid were then quickly added while shaking. At 490 nm, the absorbance of orange-yellow color was measured relative to a blank (distilled water).

### Proline determination

In accordance with Bates et al. (1973), the proline content was measured. Using a homogenizer, the tissue was extracted in 3% (w/v) sulphosalicylic acid. Whatman filter paper was used to filter the homogenate. Two milliliters of the extraction, glacial acetic acid, and ninhydrin reagent samples were heated in a test tube in a water bath at 100°C for 60 min. After cooling, 4 ml of toluene was added to the reaction mixture, which had been forcefully stirred in a test tube for 15–20 s. After being extracted from the aqueous phase, the toluene-containing chromophore was allowed to warm to room temperature. Toluene was used as a blank to measure the absorbance at 520 nm.

### Na+ and K+ determination.

Seedling samples were initially dried at 80 °C for 72 h, then pulverized using an electric grinder (Model ECG-200; Hefei Ecocoffee Co., Ltd., Hefei, China) for subsequent determination of sodium (Na⁺) and potassium (K⁺) concentrations. The samples were further dried overnight at 100 °C the day before digestion. For microwave-assisted acid digestion (Anton Paar Multiwave 5000, Graz, Austria), 0.5 g of each sample was placed in a vessel containing 10 mL of nitric acid. The digestion process involved heating to 175 °C within 5.5 min, followed by maintaining this temperature for 4.5 min. After cooling, digests were diluted to 50 mL with deionized water. Each analysis was performed using three biological replicates per treatment, with samples pooled from 20 seedlings per replicate. An FLM3 flame photometer was used to measure ions. The reference solution, kept at room temperature (25°C), contained potassium chloride (5 ± 0.5 mmol/L) and sodium chloride (14 ± 1.4 mmol/L). A blank was created by mixing 500 ml of purified water with five milliliters of concentrated lithium chloride (300 ± 5 mmol/L) (Chapman and Pratt 1962).

### Inorganic phosphorus (P) determination

Dried and ground plant samples were used for inorganic phosphorus determination. Each treatment consisted of three biological replicates, with samples pooled from 20 seedlings per replicate. A commercial kit from Quimica Clinica Aplicada S.A. (Spain) was used to detect the phosphate ion. Phosphomolybdate is formed when P and molybdate react and is subsequently reduced to molybdenum blue, which can be examined photometrically at 650 nm. Potassium dihydrogen phosphate (KH₂PO₄) was used as the reference standard (conc. 4 mg%), and zero correction was made against the reagent blank.

### Extraction for enzyme activity

Fresh seedlings (0.1–0.4 g; three biological replicates, each comprising 20 seedlings) were weighed, stored at –20 °C, and subsequently processed following the protocol described by Ni et al. (2001). In short, a cold potassium phosphate buffer (0.1 M, pH 7.0) containing 1% (w/v) polyvinylpyrrolidone and 1% (v/v) Triton X-100 was used to extract the enzymes from frozen plant samples. 1 mL of the extraction buffer was used to macerate the samples. An additional 1 mL of the extraction buffer was used to grind the samples further. For every sample, 2 mL of the extraction buffer was used. A 1.5 mL aliquot of the extract was centrifuged for 10 min at 4˚C at 10,000 g. For subsequent enzyme activity tests, the supernatant was promptly refrigerated.

### Catalase activity

Catalase analysis kit was purchased from (Biodiagnostic, kit No. CA 2517, country), which is based on the spectrophotometric approach outlined by Aebi (1984). Approximately 3 mL of reaction mixture was prepared, comprising 1.5 mL of 100 mM potassium phosphate buffer (pH 7.0), 0.5 mL of 75 mM H₂O₂, 0.05 mL enzyme extract, and distilled water. The reaction was initiated by H₂O₂ addition, and absorbance reduction was measured over 1 min. At 510 nm, the absorbance was measured.

### Peroxidase activity

Peroxidase activity was measured using Hammerschmidt et al. (1982) method. 100 μL of enzyme extract and 1.5 mL of pyrogallol (0.05 M) were put into a spectrophotometer sample cuvette. At 420 nm, the readings were set to zero. The sample cuvette was filled with 100 μL of hydrogen peroxide (1%) to start the reaction. The change in absorbance /min/g sample was used to express the enzyme activity.

### Glutathione-S-transferase activity

Glutathione S-transferase (GST) activity was assayed following the procedure of Habig et al. (1974), with modifications for plant tissue. The substrates used were 1-chloro-2,4-dinitrobenzene (CDNB) and reduced glutathione (GSH). The reaction mixture contained final concentrations of 20 mM GSH (100 μL) and 20 mM CDNB (25 μL), dissolved in 2.5% ethanol, 1 mL of 0.1 M potassium phosphate buffer (pH 6.5), and 200 μL of enzyme extract. GST activity was monitored spectrophotometrically by recording absorbance at 340 nm, using an extinction coefficient of 9.6 mM⁻^1^ cm⁻^1^ to calculate enzyme activity.

### Total antioxidant capacity

The spectrophotometric approach created by Prieto et al. (1999) was used to determine the antioxidant capacity. This assay relies on the reduction of Mo (VI) to Mo (V) by antioxidant compounds in the extract, resulting in the formation of a green phosphate/Mo (V) complex under acidic conditions. For the reaction, 0.1 mL of extract was mixed with 1 mL of reagent solution containing 0.6 M sulfuric acid, 28 mM sodium phosphate, and 4 mM ammonium molybdate. The mixtures were incubated at 95 °C for 90 min, after which they were cooled to room temperature, and the absorbance was recorded at 695 nm using a double-beam UV–visible spectrophotometer. Methanol (0.1 mL) was used in place of the extract as a blank. Antioxidant activity was expressed in terms of ascorbic acid equivalents, based on a calibration curve prepared with standard solutions of ascorbic acid (100, 50, 25, 12.5, and 6.25 μg/mL in methanol).

**Fig. 1:**
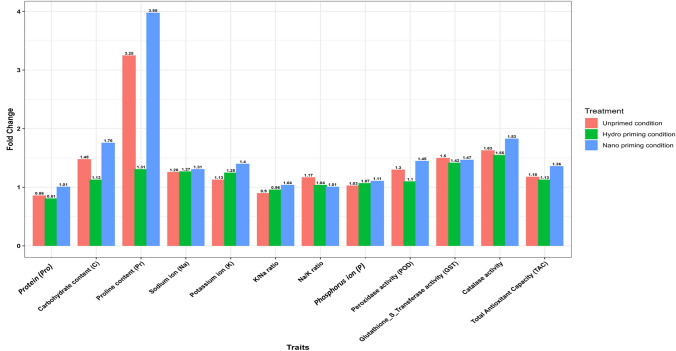
The fold change of biochemical traits calculated for the mean of all genotypes under salinity stress compared to control conditions for all traits (Protein content, Carbohydrate content, Proline content, Sodium ion, Potassium ion, K^+^/Na^+^ ratio, Na^+^/K^+^ ratio, Phosphorus content, Peroxidase activity, Glutathione_S_Transferase activity, Catalase activity, and Total Antioxidant Capacity) under all treatments (unprimed, hydro-priming, and nano-priming conditions). Values represent means of three biological replicates

Fold changes for each trait under all treatments were expressed as the mean values under salinity treatment relative to those under control conditions. Figure 1 shows fold change values calculated for the mean of all genotypes together. Fig. S2 presents fold change values for each genotype individually under each treatment.

Each value represents the mean of three biological replicates. Error bars in figures indicate the standard error of the mean (SE).

### Gene expression pattern assay

To investigate ten salinity-related genes, the total RNA was isolated from seedlings of three biological replicates (20 plants/biological replicate) of selected three tolerant and two sensitive barley genotypes using the RNeasy Plant Mini Kit (Qiagen, Valencia, CA, USA). To suppress RNases and stop RNA breakdown, all instruments needed for RNA extraction were sterilized. A Nano-Drop Spectrophotometer device (Thermo Scientific-2000C, Waltham, MA, USA) was used to find the concentration of the isolated RNA. 1% agarose gel electrophoresis was then used to evaluate the quality of the isolated total RNA. For cDNA synthesis, 1 µg of total RNA was reverse-transcribed using the Reverse Transcription Kit (Thermo Fisher Scientific, USA) according to the manufacturer’s instructions. All RT-qPCR reactions were run in a MiniOpticonTM Real-Time PCR device (Bio-Rad, Hercules, CA, USA). Each reaction contained 2 µL of cDNA, 0.5 µM of each primer, 10 µL of SYBR Green mix (QIAGEN, Germany), and nuclease-free water in a final volume of 20 µL. The cycling conditions were: initial denaturation at 95 °C for 10 min, followed by 40 cycles of 95 °C for 15 s and 60 °C for 60 s. Melt-curve analysis was performed to confirm amplification specificity.

Normalization of the expression of the studied genes was carried out using the *HvActin* gene. Then, using the 2^−∆∆CT^ relationship and the melting temperature for each primer, the relative expression levels of the genes under investigation were determined (Pfaffl 2001). The sequences and more information for primers used in the expression study are listed in Table 2.

**Table 2:** lists of gene symbols, full gene names, gene categories, and the forward and reverse primer sequences that are related to the genes

Gene symbol	Full gene name	Gene Category	Forward (F)/Reverse (R)	Sequence (5’—3’)	Reference
***HvNHX1***	**Na**^**+**^**/H**^**+**^** exchanger 1**	Ion Transporter- related genes	F	TGCATATCTACCAGTGCTTAT	Adem et al. (2014)
			R	GGTTCAAGACACAAGTTCAGT	
***HvNHX2***	**Na**^**+**^**/H**^**+**^** exchanger 2**		F	GGTTTTCGGCTTGCTGACTAA	Adem et al. (2014)
			R	CATTGGGCGCATGAACTTATC	
***HvNHX3***	**Na**^**+**^**/H**^**+**^** exchanger 3**		F	TGAGCCGAACATTACTGTGAT	Adem et al. (2014)
			R	ACGAGCTTACCTTTCAATACA	
***HvSOS1***	**Salt Overly Sensitive 1**		F	GGCACCAACAGGAAGATGAA	Yousefi Rad et al. (2019)
			R	GATATGCAGGAGGCCAGAGA	
***HvSOS3***	**Salt Overly Sensitive 3**	Ion homeostasis/signaling	F	GCTGCACCTCGAAAATCC	Yousefi Rad et al. (2019)
			R	AAACCGCTCGTCACTGCT	
***HvSODA***	**Superoxide Dismutase A**	Antioxidant-related genes	F	TGGATGGGTGTGGCTAGCTTT	Azarin et al. (2022)
			R	AGTATGCATGCTCCCAGACAT	
***HvCAT1***	**Catalase 1**		F	AACTCCGCCTACTGGACCAC	
			R	ACGTTCAGGTATGCGTTCCC	
***HvAPX***	**Ascorbate Peroxidase**		F	GAGGTCTGGCTTTGAGGGAC	
			R	TCAGCAGAGTTTTGTCACTTGGA	
***HvGR***	**Glutathione Peroxidase**		F	GAGCTACGACTACGACCTCTTC	
			R	CACGTATCACGCACGTCCC	
***HvGPX***	**Glutathione Reductase**		F	CTGATCCCGCACTACAGCTT	Cembrowska-Lech and Rybak (2023)
			R	CCCGTCCTTGAGCATCTTCC	
***HvActin***	**Actin**	Reference gene	F	CCCTAGCATAGTTGGTCGCC	Azarin et al. (2022)
			R	CTCGATGGGGTACTTGAGCG	

### Data analysis

Using PLABSTAT software (Utz 2011) and the following statistical model, the analysis of variance (ANOVA) was computed under all conditions:

$${\text{Yi j}} = \mu + {\mathrm{gi}} + {\mathrm{rj}} + {\text{gri j}}$$

gi and rj are the primary impacts of genotypes and replications, respectively; Yi j is an observation of genotype i in replication j under each circumstance; and μ is the global mean; Gr i j is a representation of the genotype × replication interaction between genotype I and replication J.

Using SRplot (Tang et al. 2023), PCA and heatmap analysis were carried out. R software version 4.4.2, packages tidyverse (dplyr and tidyr), and ggplot2 were used to manipulate and visualize the data for each parameter for gene expression.

## Results

### Biochemical responses

To assess the impact of salinity stress on contrasting genotypes (tolerant vs sensitive), biochemical traits were analyzed in five tolerant (BCC1389, BCC1416, BCC1469, BCC502, BCC538) and five sensitive genotypes (BCC1498, BCC1505, BCC173, BCC526, BCC532). Significant phenotypic variation was observed across all traits under both control (C) and salinity (S) conditions, regardless of treatment type, unprimed (UP), hydro-priming (H), or nano-priming (N). Nano-priming enhanced all traits under salinity compared to the control (Supplementary Tables S1, S2, and S3). Meanwhile, under salinity stress, protein content and the K⁺/Na⁺ ratio declined in (UP and H), while other parameters increased. This is demonstrated by the fold changes under (N) were greater than those under (H and UP) conditions, except for the Na⁺/K⁺ ratio and glutathione-S-transferase (Fig. 1).

Under salinity stress, tolerant genotypes generally showed an increase in all traits but reduced protein (Pro) and K⁺/Na⁺ ratio across all treatments. In addition, peroxidase activity and total antioxidant capacity were decreased under salinity with hydro-priming (S_H), while the phosphorus content was reduced only under salinity for unprimed conditions (S_UP) (Supplementary Fig. S1). Sensitive genotypes also showed trait increases, but Pro and K⁺/Na⁺ ratio decreased under (UP and H), while (N) reduced the Na⁺/K⁺ ratio (Supplementary Fig. S1).

The analysis of variance (ANOVA) revealed substantial genetic variation among genotypes across all traits, with a highly significant interaction between treatments (T) and genotypes (G) (Table 3). Significant differences (*p* < 0.01) were observed between control and salinity treatments, indicating that both genetic diversity and environmental conditions strongly influenced barley responses under varying growth environments.

**Table 3:** Analysis of variance (ANOVA) for all biochemical traits under unprimed (UP), hydro-priming (H), and nano-priming (N) conditions (combined ANOVA)

Sources of variance	Genotypes (G)			Treatment (T)			Replicates (R)			T x G			R x G		
	UP	H	N	UP	H	N	UP	H	N	UP	H	N	UP	H	N
(Degree of freedom) DF	9			1			2			9			18		
Protein (Pro)	147.62^**^	258.51^**^	550.35^**^	145.28^**^	269.34^**^	1.97	0.45	0.16	2.84^+^	42.45^**^	34.44^**^	84.67^**^	1.07	1.05	1.16
Carbohydrates (C)	406.18^**^	582.56^**^	1126.91^**^	1532.87^**^	170.56^**^	5092.82^**^	0.27	0.93	2.22	42.72^**^	41.19^**^	157.22^**^	1.19	0.71	2.43*
Proline (Pr)	286.98^**^	1288.55^**^	510.80^**^	4859.33^**^	403.38^**^	3484.71^**^	0.15	4.84^*^	2.73^+^	241.71^**^	456.97^**^	325.09^**^	1.25	2.67*	1.54
Sodium (Na^+^)	625.78^**^	717.85^**^	637.20^**^	2837.84^**^	1898.74^**^	2220.59^**^	0.62	2.31	3.61^*^	62.68^**^	46.16^**^	81.51^**^	1.22	1.03	2.17*
Potassium (K^+^)	491.24^**^	493.76^**^	223.11^**^	146.47^**^	366.25^**^	350.50^**^	0.00	0.61	0.49	200.29^**^	66.27^**^	87.94^**^	1.10	0.48	0.46
Potassium- Sodium ratio (K^+^/Na^+^)	157.87^**^	175.77^**^	130.65^**^	35.22^**^	2.02	2.52	0.00	0.00	2.25	73.73^**^	36.88^**^	48.71^**^	0.75	0.70	0.00
Sodium- Potassium ratio (Na^+^/K^+^)	78.07^**^	227.62^**^	117.07^**^	86.89^**^	8.12^**^	0.11	0.05	0.59	0.69	57.98^**^	33.57^**^	31.14^**^	0.68	0.62	0.41
Phosphorus (P)	111.57^**^	429.86^**^	205.02^**^	16.87^**^	232.47^**^	376.22^**^	0.87	0.02	1.64	10.92^**^	32.84^**^	18.92^**^	0.43	2.77*	1.64
Peroxidase (POD)	1258.71^**^	1186.13^**^	1342.80^**^	937.57^**^	106.01^**^	1935.46^**^	2.10	0.84	3.07^+^	293.63^**^	104.98^**^	190.50^**^	1.30	1.36	1.41
Glutathione S Transferase (GST)	248.89^**^	475.04^**^	1620.01^**^	1681.71^**^	1486.95^**^	4327.71^**^	1.26	0.52	0.57	165.79^**^	89.58^**^	312.57^**^	1.08	0.87	2.11^+^
Catalase (CAT)	355.69^**^	2374.55^**^	885.41^**^	2283.21^**^	8693.77^**^	4995.56^**^	2.19	5.18^*^	0.57	170.51^**^	828.87^**^	374.07^**^	0.54	5.87^**^	1.16
Total Antioxidant Capacity (TAC)	741.52^**^	916.00^**^	646.43^**^	685.18^**^	324.87^**^	1001.10^**^	3.18 +	1.93	0.60	133.69^**^	176.07^**^	56.18^**^	2.30^*^	1.30	1.09

^*^significant at P = 0.05 and + significant at P = 0.1

^**^Significant at P = 0.01

### Protein content (Pro)

**Fig. 2:**
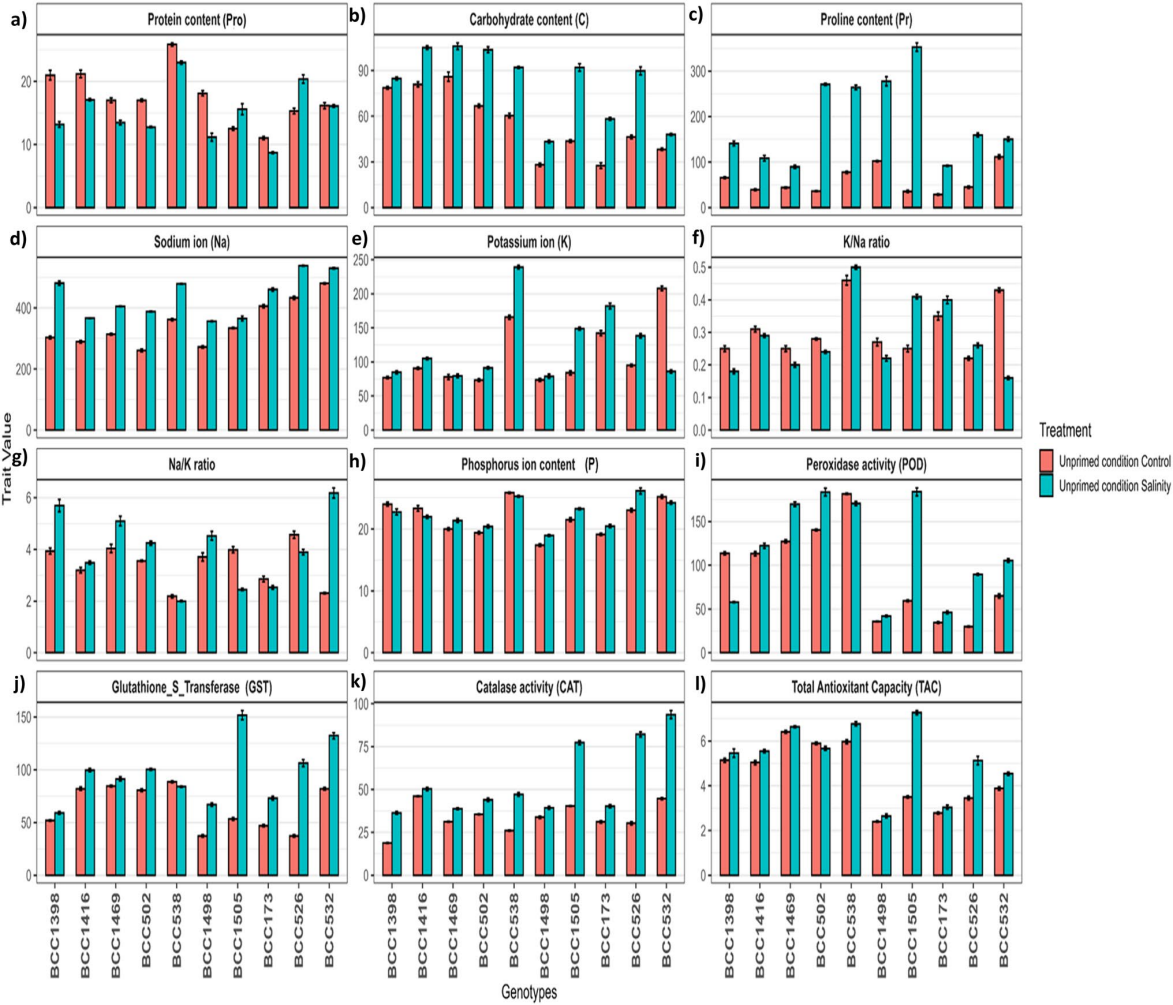
Biochemical changes in tolerant and sensitive genotypes under control and salinity for all traits under unprimed conditions **a** Protein content **b** Carbohydrate content **c** Proline content **d** Sodium ion **e** Potassium ion **f** K^+^/Na^+^ ratio **g** Na^+^/K^+^ ratio **h** Phosphorus content **i** Peroxidase activity **j** Glutathione_S_Transferase activity **k** Catalase activity **l** Total Antioxidant Capacity. The five tolerant genotypes are on the left side, and the five sensitive genotypes are on the right side. Values represent means ± SE of three biological replicates

**Fig. 3:**
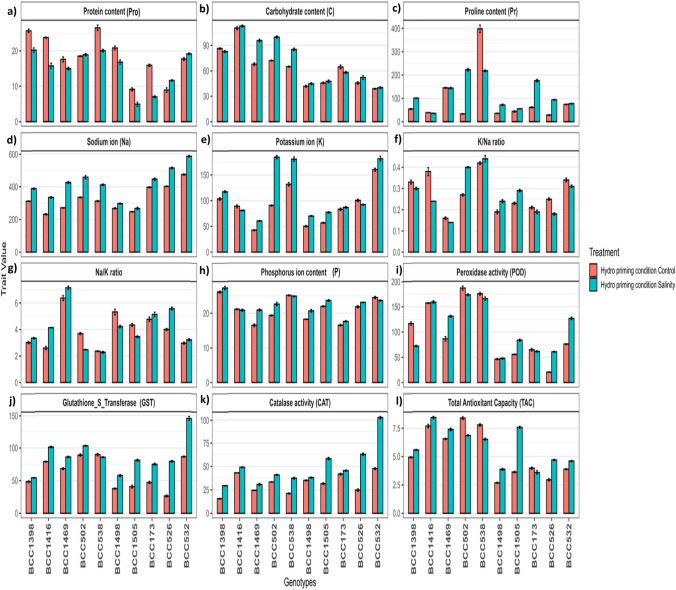
Biochemical changes in tolerant and sensitive genotypes under control and salinity for all traits under hydro-priming conditions **a** Protein content **b** Carbohydrate content **c** Proline content **d** Sodium ion **e** Potassium ion **f** K^+^/Na^+^ ratio **g** Na^+^/K^+^ ratio **h** Phosphorus content **i** Peroxidase activity **j** Glutathione_S_Transferase activity **k** Catalase activity **l** Total Antioxidant Capacity. The five tolerant genotypes are on the left side, and the five sensitive genotypes are on the right side. Values represent means ± SE of three biological replicates

**Fig. 4:**
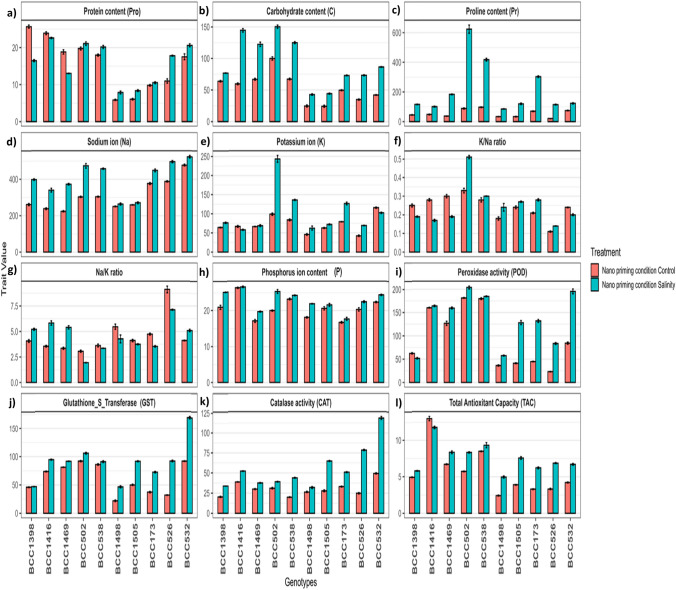
Biochemical changes in tolerant and sensitive genotypes under control and salinity for all traits under nano-priming conditions **a** Protein content **b** Carbohydrate content **c** Proline content **d** Sodium ion **e** Potassium ion **f** K^+^/Na^+^ ratio **g** Na^+^/K^+^ ratio **h** Phosphorus content **i** Peroxidase activity **j** Glutathione_S_Transferase activity **k** Catalase activity **l** Total Antioxidant Capacity. The five tolerant genotypes are on the left side and the five sensitive genotypes on the right side. Values represent means ± SE of three biological replicates

Protein content for nano-priming showed the strongest increase under salinity (1.01-fold), unprimed (0.86-fold), and hydro-priming (0.81-fold) (Fig. 1). Under (UP) conditions, Pro content generally declined under salinity (Fig. 2a). The BCC538 (tolerant) and BCC526 (sensitive) showing the strongest fold change, while BCC1398 and BCC1498 had the lowest (Supplementary Fig. S2). Under (H) conditions, most genotypes showed reduced Pro under salinity (Fig. 3a). The BCC502 (tolerant) and BCC526 (sensitive) had the highest fold increases, while BCC1416 and BCC173 were the lowest (Supplementary Fig. S2). Under (N) conditions, Pro content increased under salinity in most genotypes (Fig. 4a). The BCC538 (tolerant) and BCC526 (sensitive) showed the greatest fold changes, while BCC1398 and BCC173 had the lowest (Supplementary Fig. S2).

### Carbohydrate content (C)

Carbohydrate content under nano-priming showed the strongest increase under salinity (1.76-fold), unprimed (1.48-fold), and hydro-priming (1.13-fold) (Fig. 1).

Under (UP) conditions, carbohydrate content increased under salinity in all genotypes (Fig. 2b). The BCC502 (tolerant) and BCC173 (sensitive) showed the highest fold changes (Supplementary Fig. S2), whereas BCC1398 and BCC532 had the lowest (Supplementary Fig. S2). Under (H) conditions, most genotypes exhibited increased carbohydrate content under salinity, except BCC1398 (tolerant) and BCC173 (sensitive) (Fig. 3b). BCC1469 and BCC526 had the highest fold changes, while BCC1398 and BCC173 were the lowest responders (Supplementary Fig. S2). Under (N) conditions, carbohydrate content increased under salinity in all genotypes (Fig. 4b). The BCC1416 (tolerant) and BCC526 (sensitive) showed the strongest fold changes, while BCC1398 and BCC173 had the weakest (Supplementary Fig. S2).

### Proline content (Pr)

Proline content under nano-priming showed the strongest increases under salinity (3.98-fold), unprimed (3.25-fold), and hydro-priming (1.31-fold) (Fig. 1). Under (UP) conditions, Pr increased under salinity in all genotypes (Fig. 2c), with BCC502 (tolerant) (7.45-fold) and BCC1505 (sensitive) (9.89-fold) showing the highest fold changes (Supplementary Fig. S2), while BCC1469 (tolerant) (2.03-fold) and BCC532 (sensitive) (1.34-fold) had the lowest (Supplementary Fig. S2). Under (H) conditions, Pr decreased in most tolerant genotypes but increased in all sensitive ones (Fig. 3c). BCC502 (tolerant) (6.68-fold) and BCC526 (sensitive) (3.31-fold) had the highest fold changes, while BCC538 (tolerant) (0.54-fold) and BCC532 (sensitive) (1.04-fold) were the lowest (Supplementary Fig. S2). Under (N) conditions, Pr increased under salinity in all genotypes (Fig. 4c), with BCC502 (tolerant) (7.06-fold) and BCC526 (sensitive) (5.5-fold) showing the strongest fold changes, while BCC1416 (2.09-fold) and BCC532 (sensitive) (1.65-fold) had the lowest (Supplementary Fig. S2).

### Minerals (sodium, potassium, and phosphorus) related traits

Sodium levels increased under salinity in all genotypes (Figs. 2d, 3d, and 4d). Nano-priming minimized sodium content, followed by hydro-priming and unprimed seeds (Supplementary Tables S1, S2, and S3). Compared to control, salinity increased the Na^+^ content by 1.31-fold with (N), 1.27-fold with (H), and 1.26-fold with (UP) (Fig. 1). Under (UP) conditions, BCC1398 (tolerant) and BCC1498 (sensitive) showed the highest fold changes (1.59 and 1.30-fold changes, respectively) (Supplementary Fig. S2), while BCC1416 (tolerant) and BCC1505 (sensitive) had the lowest (1.27 and 1.09-fold changes, respectively) (Supplementary Fig. S2). Under (H), BCC1469 (tolerant) and BCC526 (sensitive) showed the highest fold changes (1.57 and 1.28-fold changes, respectively), while BCC1398 (tolerant) and BCC1505 (sensitive) were the lowest responders (1.25 and 1.08-fold changes, respectively) (Supplementary Fig. S2). Under (N), BCC1469 (tolerant) and BCC526 (sensitive) showed the strongest fold changes (1.66 and 1.28-fold changes, respectively), while BCC1416 (tolerant) and BCC1505 (sensitive) had the weakest (1.43 and 1.04-fold changes, respectively) (Supplementary Fig. S2).

Surprisingly, Potassium content increased under salinity in all genotypes except for BCC532 (sensitive) under unprimed conditions, BCC1416 (tolerant) and BCC526 (sensitive) under hydro-priming, and BCC1416 (tolerant) and BCC532 (sensitive) under nano-priming (Figs. 2e, 3, 4e). Under control and salinity, (N) showed the lowest K^+^ content, followed by (H and UP) conditions (Supplementary Tables S1, S2, and S3). The increment under salinity compared to control was 1.40-fold under (N), 1.25-fold under (H), and 1.13-fold under (UP) conditions (Fig. 1).

Under (UP) conditions, BCC538 (tolerant) and BCC1505 (sensitive) showed the highest fold changes (1.44 and 1.77-fold changes, respectively) (Supplementary Fig. S2), while BCC1469 (tolerant) and BCC532 (sensitive) had the lowest (1.02 and 0.41-fold changes, respectively) (Supplementary Fig. S2). Under (H), BCC502 (tolerant) and BCC1498 (sensitive) showed the highest fold changes (2.04 and 1.04-fold changes, respectively), while BCC1416 (tolerant) and BCC526 (sensitive) were the lowest responders (0.91 and 0.92-fold changes, respectively) (Supplementary Fig. S2). Under (N), BCC502 (tolerant) and BCC526 (sensitive) showed the strongest fold changes (2.46 and 1.63-fold changes, respectively), while BCC1416 (tolerant) and BCC532 (sensitive) had the lowest (0.87 and 0.89-fold changes, respectively) (Supplementary Fig. S2).

The K^+^/Na^+^ ratio, under control and salinity, the nano-priming showed the low K^+^/Na^+^ ratio followed by hydro-priming and unprimed conditions (Supplementary Tables S1, S2, and S3). The increment under salinity compared to control was 1.04-fold under nano-priming, 0.96-fold under hydro-priming, and 0.9-fold under unprimed conditions (Fig. 1).

For the Na^+^ /K^+^ ratio, under control and salinity conditions, the nano-priming showed the highest Na^+^ /K^+^ ratio, followed by hydro-priming and unprimed conditions (Supplementary Tables S1, S2, and S3). The increment under salinity compared to control was 1.01-fold under nano-priming, 1.04-fold under hydro-priming, and 1.17-fold under unprimed conditions (Fig. 1).

For the phosphorus content (P), under salinity, phosphorus was higher than in the control (Supplementary Tables S1, S2, and S3). Under control, unprimed conditions showed the highest P content, followed by nano and hydro-priming conditions; meanwhile, under salinity, the nano-priming (N) showed the highest P content, followed by hydro-priming and unprimed conditions (Supplementary Tables S1, S2, and S3). Under salinity, the increment in phosphorus compared to control was 1.03-fold under (UP) conditions, 1.07-fold under (H), and 1.11-fold under (N) (Fig. 1).

### Enzyme activity (peroxidase, glutathione S transferase, and catalase)

Peroxidase (POD) activity was highest under hydro-priming in the control and under nano-priming in salinity (Supplementary Tables S1, S2, and S3). Salinity increased POD activity by 1.3-fold in unprimed, 1.1-fold in hydro-priming, and 1.45-fold in nano-priming (Fig. 1). POD activity under salinity varies across treatments (Figs. 2i, 3i, and 4i). Under (UP) conditions, BCC1469 (tolerant) and BCC1505 (sensitive) showed the highest fold changes, while BCC1398 (tolerant) and BCC1498 (sensitive) had the lowest (Supplementary Fig. S2). Under (H), BCC1469 (tolerant) and BCC526 (sensitive) showed the highest fold changes, while BCC1398 (tolerant) and BCC173 (sensitive) were the lowest responders (Supplementary Fig. S2). Under (N), BCC1469 (tolerant) and BCC526 (sensitive) showed the strongest fold changes, while BCC1398 (tolerant) and BCC1498 (sensitive) had the weakest (Supplementary Fig. S2).

Glutathione S-transferase activity (GST) was highest in unprimed treatments, followed by nano and hydro under salinity (Supplementary Tables S1, S2, and S3). Salinity increased GST activity by 1.5-fold in unprimed, 1.42-fold in hydro-priming, and 1.47-fold in nano-priming (Fig. 1). GST activity under salinity varied across treatments (Figs. 2j, 3j, and 4j). Under (UP) conditions, BCC502 (tolerant) and BCC526 (sensitive) showed the highest fold changes, while BCC538 (tolerant) and BCC173 (sensitive) had the lowest (Supplementary Fig. S2). Under (H), BCC1416 (tolerant) and BCC526 (sensitive) showed the highest fold changes, while BCC538 (tolerant) and BCC1498 (sensitive) were the lowest responders (Supplementary Fig. S2). Under (N), BCC1416 (tolerant) and BCC526 (sensitive) showed the strongest fold changes, while BCC1398 (tolerant) and BCC1505 (sensitive) had the lowest changes (Supplementary Fig. S2).

Catalase activity (CAT) was highest in nano-priming under salinity (Supplementary Tables S1, S2, and S3). Salinity increased activity by 1.63-fold in unprimed, 1.55-fold in hydro-priming, and 1.83-fold in nano-priming (Fig. 1). CAT activity under salinity increased in all genotypes (Figs. 2k, 3k, and 4k). Under (UP) conditions, BCC1398 (tolerant) and BCC526 (sensitive) showed the highest fold changes, while BCC1416 (tolerant) and BCC1498 (sensitive) had the lowest (Supplementary Fig. S2). Under (H), BCC1398 (tolerant) and BCC526 (sensitive) showed the highest fold changes, while BCC1416 (tolerant) and BCC1498 (sensitive) were the lowest responders (Supplementary Fig. S2). Under (N), BCC538 (tolerant) and BCC526 (sensitive) showed the strongest fold changes, while BCC1469 (tolerant) and BCC1498 (sensitive) had the weakest (Supplementary Fig. S2).

Total antioxidant capacity was highest with nano-priming under both control and salinity, followed by hydro and unprimed (Supplementary Tables S1, S2, and S3). Salinity increased capacity by 1.36-fold in nano-priming, 1.18-fold in unprimed, and 1.13-fold in hydro-priming (Fig. 1). Total antioxidant capacity (TAC) under salinity varied across treatments (Figs. 2l, 3l, and 4l). Under (UP) conditions, BCC538 (tolerant) and BCC1505 (sensitive) showed the highest fold changes, while BCC502 (tolerant) and BCC173 (sensitive) had the lowest (Supplementary Fig. S2). Under (H), BCC1398 (tolerant) and BCC1505 (sensitive) showed the highest fold changes, while BCC502 (tolerant) and BCC173 (sensitive) were the lowest responders (Supplementary Fig. S2). Under (N), BCC502 (tolerant) and BCC526 (sensitive) showed the strongest fold changes, while BCC1416 (tolerant) and BCC532 (sensitive) had the weakest (Supplementary Fig. S2).

### Principal component analysis

Principal component analysis (PCA) was performed to explore the variation in biochemical traits under control and salinity across all treatments (UP, H, and N) (Supplementary Fig. S3).

Under UP, 59.2% of the variation was explained, with PC1 and PC2 accounting for 36.7% and 22.5%, respectively (Supplementary Fig. S3a). The biplot showed a clear separation between control for unprimed conditions (C_UP) and salinity for unprimed conditions S_UP. Greater K⁺ and the K⁺/Na⁺ ratio were associated with C_UP, indicating improved ionic equilibrium. In contrast, S_UP clustered with stress-related traits such as proline (Pr), phosphorus (P), catalase (CAT), glutathione-S-transferase (GST), peroxidase (POD), carbohydrates (C), and total antioxidant capacity (TAC). Total protein (Pro) and sodium (Na⁺) were located in the overlapping region, reflecting intermediate variance. PC1 primarily represented ionic imbalance and antioxidant activation, while PC2 captured secondary variation in protein and nutrient metabolism. A negative correlation was observed between the K⁺/Na⁺ ratio and the Na⁺/K⁺ ratio, and a positive correlation between K⁺ and the K⁺/Na⁺ ratio.

Under H, 60.9% of the variance was explained (PC1 = 39.8%, PC2 = 21.1%) (Supplementary Fig. S3). The control with hydro-priming (C_H) and salinity with hydro-priming (S_H) treatments partially overlapped, suggesting similar trait responses with minor separation along PC1. Most features clustered in the overlapping region, indicating comparable performance. However, GST, CAT, Na⁺, and K⁺ were positioned within the S_H cluster, highlighting their stronger contribution under salinity. This pattern suggests salinity mainly influenced antioxidant activity (CAT, GST) and ionic balance (Na⁺, K⁺), while other biochemical traits remained relatively stable. A negative correlation was observed between the K⁺/Na⁺ ratio and the Na⁺/K⁺ ratio.

Under N, 66.2% of the variation was explained, with PC1 and PC2 accounting for 47.2% and 19%, respectively (Supplementary Fig. S3). Trait vectors were concentrated within the salinity with nano-priming (S_N) cluster, indicating salinity was the dominant factor driving variation. No traits were located within the control with nano priming (C_N) cluster, showing its limited impact. The distribution confirmed salinity with nano-priming (S_N) as the primary driver of variation, though some traits (Pr, K⁺, Pro, TAC) were positioned near the C_N boundary, suggesting partial responsiveness to control conditions. A negative correlation was observed between the K⁺/Na⁺ ratio and the Na⁺/K⁺ ratio.

### Heatmap of bidirectional clustering for all traits in both control and salinity conditions under all treatments

The two-way co-cluster matrix using a hierarchical co-clustering dendrogram is displayed in Supplementary Fig. S4. Row clusters were recorded at the trait or marker level across all combined data of barley genotypes germinated under various treatments, while column clusters were obtained at the genotype level. Under C_UP, the sensitive genotypes BCC526 and BCC1505 were grouped together, while the tolerant genotypes BCC502 and BCC1469 clustered together, showing close similarity. The tolerant genotype BCC538 and the sensitive genotype BCC532 were also grouped together, indicating overlapping responses. Under S_UP, the sensitive genotypes BCC173 and BCC1498, BCC526 and BCC532, and the tolerant genotypes BCC1469 and BCC1416 formed distinct clusters. Additionally, BCC538 (tolerant) and BCC1505 (sensitive) were grouped together. At the trait level, POD and TAC, Pro and P, and K⁺ and K⁺/Na⁺ clustered together under both C_UP and S_UP.

Under C_H, the tolerant genotypes BCC502 and BCC1416 and the sensitive genotypes BCC526 and BCC173 clustered together, while BCC1498 and BCC1505 (sensitive) formed another group. Under S_H, the tolerant genotypes BCC1469 and BCC1416, BCC538 and BCC502, and the sensitive genotypes BCC526 and BCC173 clustered together. At the trait level, POD and TAC clustered under C_H, while under S_H, K⁺ and K⁺/Na⁺, C and TAC, and GST and CAT formed distinct clusters.

Under C_N and S_N, the tolerant genotypes BCC502 and BCC538 and the sensitive genotypes BCC1498 and BCC1505 clustered together, showing close similarity in response patterns. Under C_N, BCC1469 and BCC1398 (tolerant) were grouped together, while BCC1398 (tolerant) and BCC526 (sensitive) also clustered, suggesting partial overlap. Similarly, BCC1469 (tolerant) and BCC173 (sensitive) were grouped together, indicating partial similarity under salinity stress. At the trait level, K⁺ and Pr clustered under both control and salinity. Additionally, P and TAC, POD and GST, Pro and C, and CAT and Na⁺ clustered under C_N, while under S_N, Pro and P, C and TAC, and GST and CAT clustered together.

### Differential gene expression in the most tolerant and sensitive genotypes

**Fig. 5:**
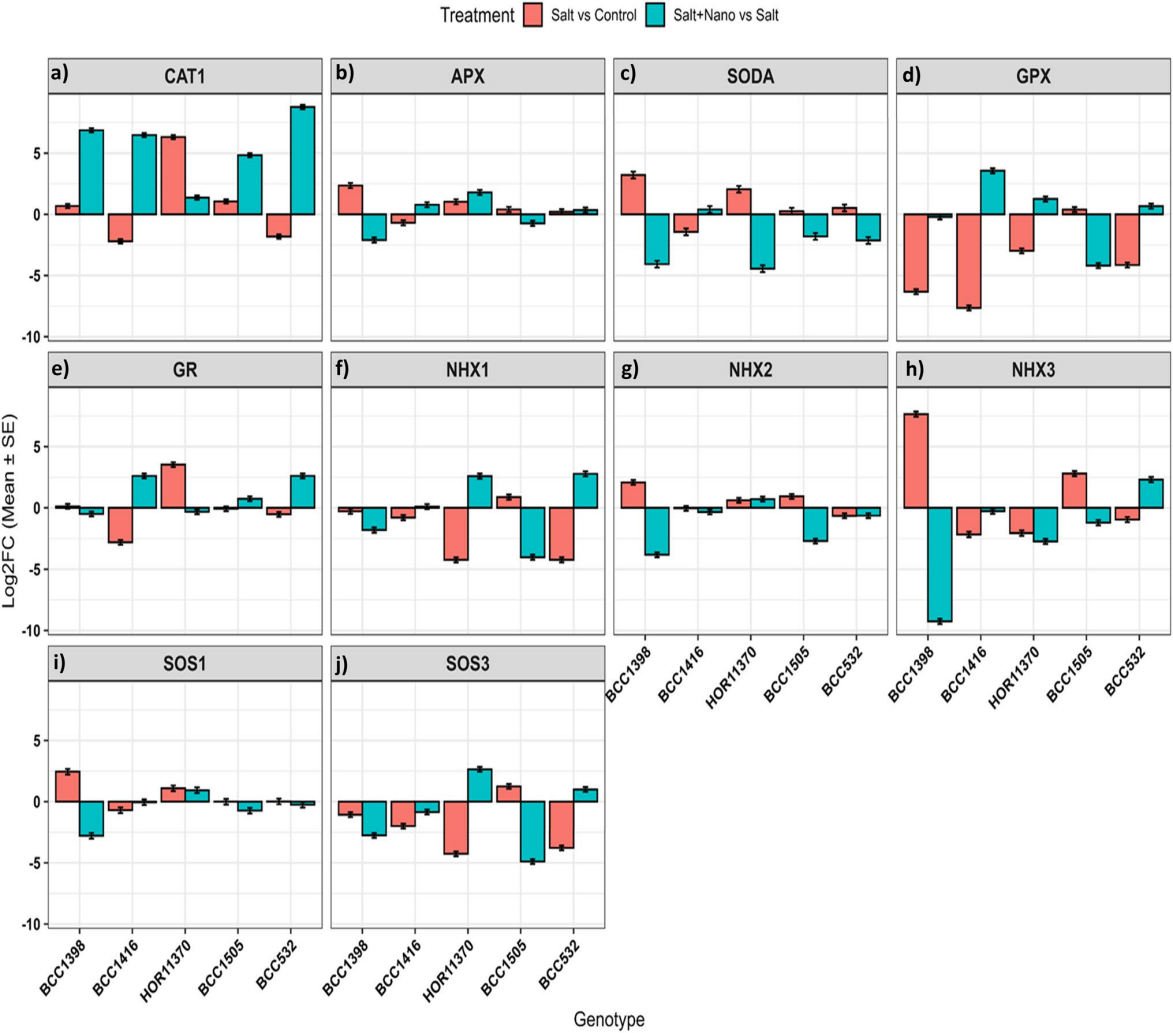
The logarithmic twofold change of all genes in five contrasting barley genotypes: BCC1398, BCC1416, HOR11370 “tolerant”, BCC1505, and BCC532 “sensitive”. X-axis, genotypes, and y-axis Log2FC. SE refers to standard error. The orange column represents the expression under salinity relative to the control, whereas the blue column represents the expression under salinity with nano relative to salt

Five contrasting genotypes, three tolerant (BCC1398, BCC1416, HOR11370) and two sensitive (BCC532, BCC1505) (Table 1) were analyzed for the expression of ten ion transporter and antioxidant-related genes under control (0 mM NaCl), salinity (200 mM NaCl), and salinity plus ZnO nanoparticle priming (200 mM NaCl + 100 ppm ZnO_NPs) at the seedling stage (Table 2). Gene expression varied across treatments and genotypes, with some showing downregulation or no change, while others were upregulated under salt stress and nano-priming (Fig. 5; Supplementary Fig. S5). In BCC1398 (tolerant) and BCC1505 (sensitive), most genes were upregulated under salinity relative to control, but downregulated with nano-priming compared to salinity (Fig. 5). In BCC1416 (tolerant) and BCC532 (sensitive), most genes were downregulated under salinity relative to control but upregulated with nano-priming relative to salinity. In HOR11370 (tolerant), most genes were upregulated under both salinity relative to control and nano-priming relative to salinity (Fig. 5).

Under salinity stress relative to the control, most genes showed low to moderate fold changes. Among antioxidant-related genes, *CAT1* was most strongly induced, with a 79.02-fold increase in tolerant HOR11370, 2.07-fold in sensitive BCC1505, and 1.62-fold in tolerant BCC1398 (Fig. 5a; Supplementary Fig. S5). *APX* was generally upregulated, highest in tolerant BCC1398 (5.13-fold) and HOR11370 (2.07-fold), but downregulated in tolerant BCC1416; sensitive genotypes showed modest increases (BCC1505: 1.31-fold; BCC532: 1.18-fold) (Fig. 5b; Supplementary Fig. S5). *SODA* was strongly induced in tolerant BCC1398 (9.44-fold) and HOR11370 (4.22-fold), but only modestly in sensitive BCC1505 (1.19-fold) and BCC532 (1.46-fold) (Fig. 5c). *GPX* was consistently downregulated, except for slight induction in BCC1505 (1.32-fold) (Fig. 5d). *GR* was downregulated in most genotypes but modestly upregulated in tolerant BCC1398 (1.11-fold) and strongly in HOR11370 (11.76-fold) (Fig. 5e). Ion transport-related genes showed subclass-specific responses. *NHX1* was downregulated in all genotypes except BCC1505 (1.84-fold) (Fig. 5f). *NHX2* was upregulated in tolerant BCC1398 (4.30-fold), HOR11370 (1.56-fold), and sensitive BCC1505 (1.91-fold), but downregulated in others (Fig. 5g). *NHX3* showed exceptionally high induction in tolerant BCC1398 (208.16-fold) and sensitive BCC1505 (6.97-fold), while other genotypes were downregulated (Fig. 5h). *SOS1* was upregulated in tolerant BCC1398 (5.62-fold), HOR11370 (2.14-fold), and sensitive BCC532 (1.05-fold) (Fig. 5i). *SOS3* was downregulated in most genotypes except BCC1505 (2.36-fold) (Fig. 5j).

Under salinity with nano-priming relative to salinity, most genes showed low to moderate fold changes, except *CAT1*, which was strongly induced in sensitive BCC532 (435.75-fold), tolerant BCC1398 (116.52-fold), and BCC1416 (88.84-fold), with moderate increases in BCC1505 (28.83-fold) and HOR11370 (2.63-fold) (Fig. 5a; Supplementary Fig. S5). *APX* was upregulated in HOR11370 (3.45-fold), BCC1416 (1.72-fold), and BCC532 (1.25-fold), but downregulated in BCC1398 and BCC1505 (Fig. 5b). *SODA* was generally downregulated, except for slight induction in BCC1416 (1.42-fold) (Fig. 5c). *GPX* was strongly induced in BCC1416 (11.64-fold), HOR11370 (2.38-fold), and BCC532 (1.55-fold), but downregulated in BCC1398 and BCC1505 (Fig. 5d). *GR* was upregulated in BCC1416 (6.03-fold), BCC532 (5.96-fold), and BCC1505 (1.65-fold), but downregulated in other genotypes (Fig. 5e).

For ion transporters, *NHX1* was upregulated in all genotypes except BCC1398 and BCC1505, with strongest induction in BCC532 (6.69-fold) and HOR11370 (6.05-fold), followed by BCC1416 (1.06-fold) (Fig. 5f). *NHX2* was mostly downregulated, except for a modest increase in HOR11370 (1.65-fold) (Fig. 5g). *NHX3* was downregulated in most genotypes but strongly induced in BCC532 (4.96-fold) (Fig. 5h). *SOS1* was downregulated in all genotypes except HOR11370 (1.9-fold) (Fig. 5i), while *SOS3* was upregulated in HOR11370 (6.30-fold) and BCC532 (1.94-fold) (Fig. 5j).

Analysis of variance (ANOVA) revealed significant genetic variation among genotypes for all genes (Supplementary Table S4).

### Relationships between gene expression data and biochemical features

**Fig. 6:**
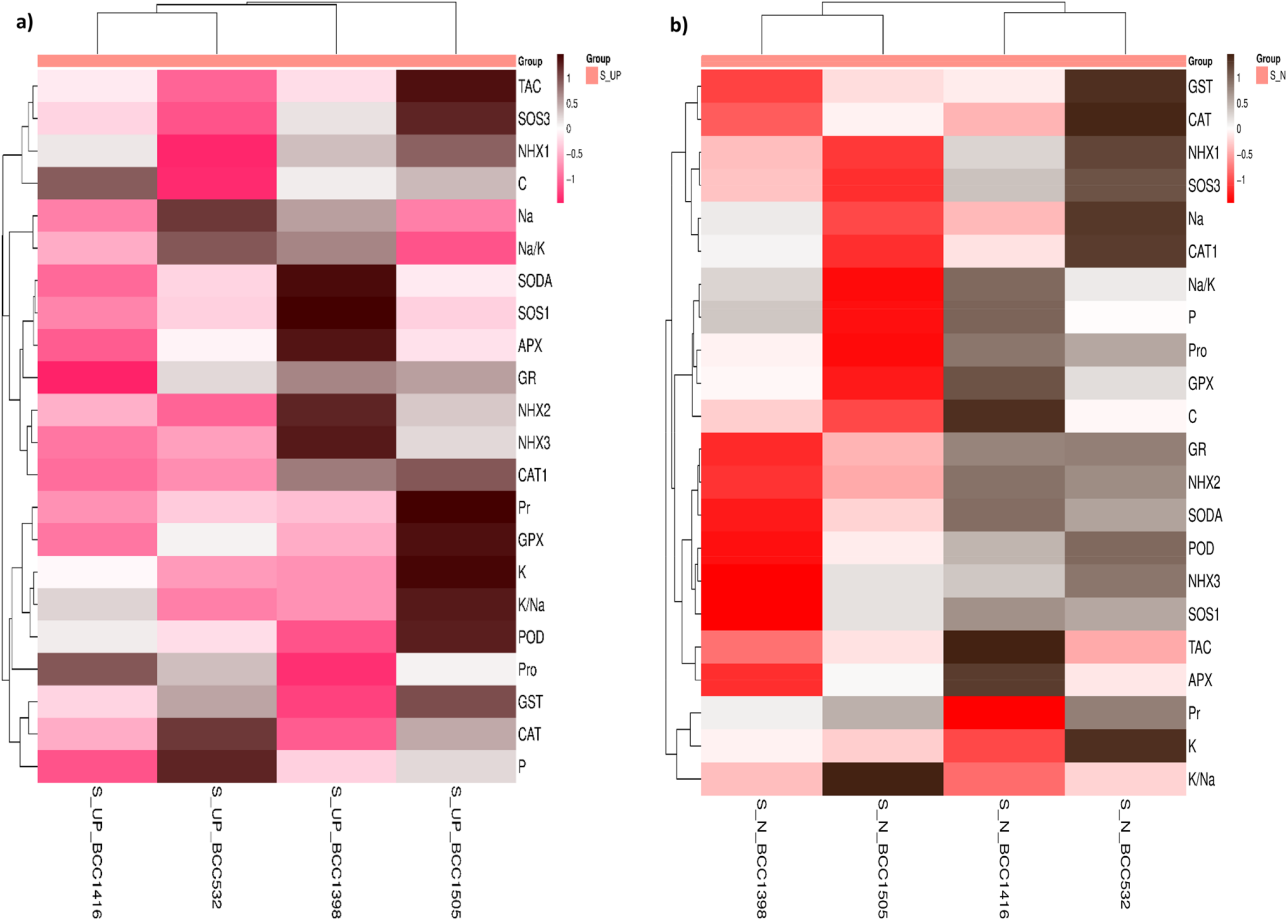
Clustering heatmap of measured characteristics and relative gene expression data in the chosen 4 barley genotypes; **a** under salinity (S) stress conditions under unprimed (UP) and **b** salinity under nano-priming (N). Where, Pro, C, Pr, Na, K, K/Na, Na/K, P, POD, GST, CAT, and TAC refer to protein, carbohydrate, proline, sodium, potassium, potassium/sodium ratio, sodium/ potassium ratio, phosphorus, peroxidase, glutathione-s-transferase, catalase, and total antioxidant capacity. The APX, CAT1, GPX, GR, NHX1, NHX2, NHX3, SODA, SOS1, and SOS3 are the expressions of those genes

Heatmap-based hierarchical cluster analysis (HCA) was performed to explore the associations between biochemical attributes and the relative expression of salt tolerance-related genes. The analysis revealed two distinct clusters of traits (Fig. 6).

Under salinity stress without priming, the clustering pattern shifted, with the sensitive genotype BCC532 grouped alongside the tolerant genotype BCC1416, indicating stress-induced convergence in biochemical performance and partial adaptation of sensitive genotypes. The remaining two genotypes formed separate clusters. At the parameter level, one cluster contained all genes except *GPX*. GST and CAT are clustered together.

Under salinity with nano-priming, a similar pattern was observed: BCC1505 (sensitive) clustered with BCC1398 (tolerant), while the remaining genotypes formed a distinct cluster. At the parameter level, one cluster contained all genes and biochemical parameters except Pr, K, and the K^+^/Na^+^ ratio. GST and CAT are clustered together.

**Fig. 7:**
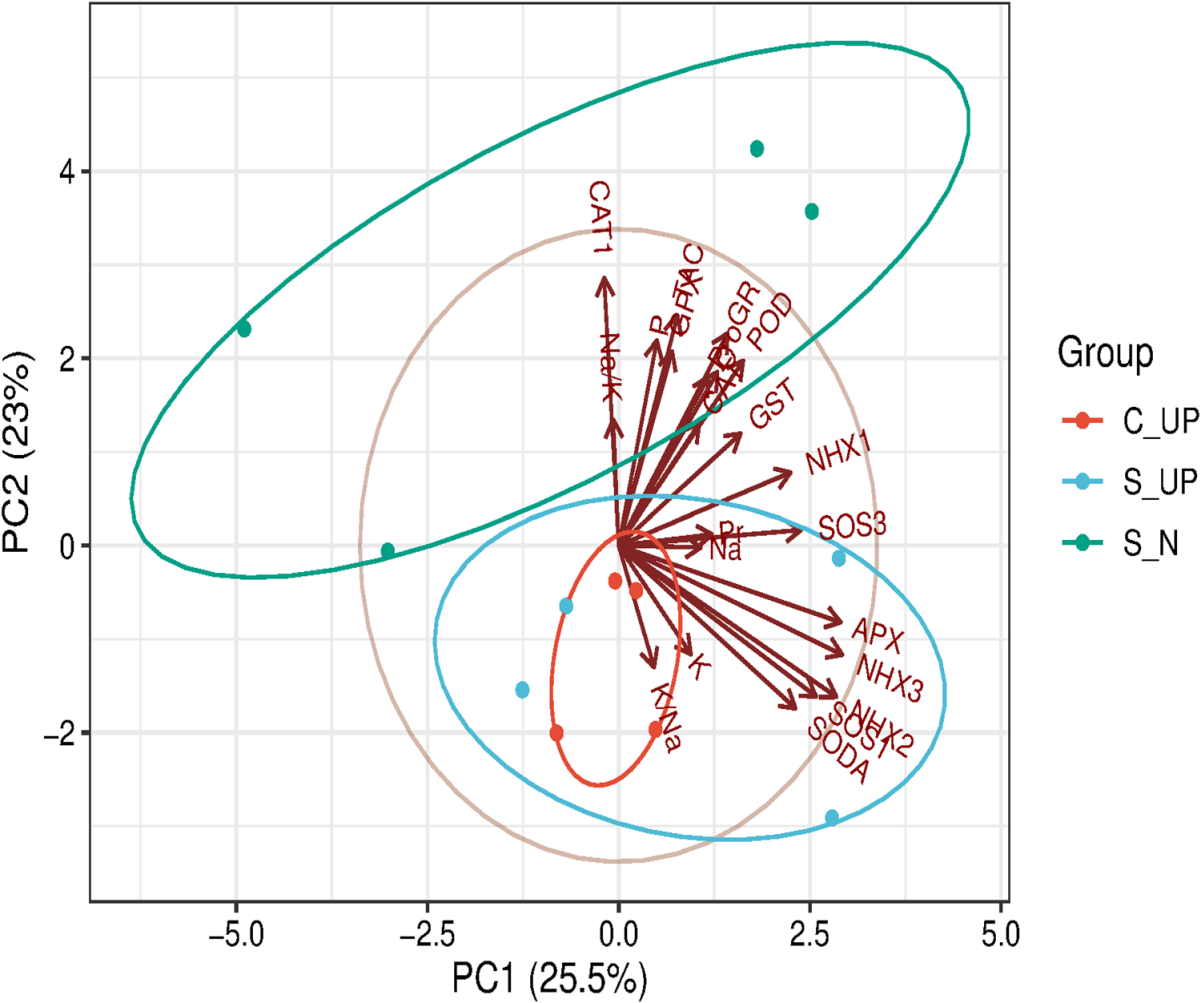
Plot of measured characteristics and relative gene expression data in the chosen 4 barley genotypes under control (C) and salinity (S) stress conditions under unprimed (UP) and salinity under nano-priming (N)using principal component analysis (PCA). Where, Pro, C, Pr, Na, K, K/Na, Na/K, P, POD, GST, CAT, and TAC refer to protein, carbohydrate, proline, sodium, potassium, potassium/sodium ratio, sodium/ potassium ratio, phosphorus, peroxidase, glutathione-s-transferase, catalase, and total antioxidant capacity. The APX, CAT1, GPX, GR, NHX1, NHX2, NHX3, SODA, SOS1, and SOS3 are the expressions of those genes

Principal component analysis (PCA) further validated these groupings, with PC1 and PC2 together explaining 48.5% of the variation. The PCA biplot confirmed the trait clustering patterns observed in HCA, strengthening the evidence for genotype-specific associations between biochemical features and gene expression (Fig. 7).

## Discussion

### Biochemical responses

Abiotic stress is a major environmental challenge that reduces arable land and leads to significant crop losses worldwide. Among these stresses, salinity is particularly critical as it interferes with water uptake during germination, thereby disrupting the imbibition process and negatively affecting seed germination (Othman et al. 2006). Crop plants exhibit adverse morpho‑physio‑biochemical responses under salt stress. These unfavorable effects arise from nutritional imbalances, metabolic disturbances, and cell membrane rupture, all of which compromise plant growth and development (Alsamadany et al. 2023).

A selected panel of eleven barley accessions (six tolerant and five sensitive genotypes) was examined for salinity responses during the early development and germination under three priming conditions: unprimed (UP), hydro-primed (H), and nano-primed (N, 100 ppm of ZnO nanoparticles). Biochemical traits assessed included protein (Pro), carbohydrates (C), proline (Pr), mineral contents (sodium (Na^+^), potassium (K^+^), phosphorus (P), the activities of antioxidant enzymes peroxidase (POD), catalase (CAT), and glutathione-S-transferase (GST), and total antioxidant capacity (TAC). In addition, the expression patterns of key stress-responsive genes were analyzed to provide molecular insights into tolerance mechanisms. Significant differences were observed between control and salinity across all treatments (UP, H, and N), confirming that the genotypes were affected by salt stress. Distinct responses among genotypes were evident in the measured traits, as demonstrated by the significance of the treatment × genotype (T × G) interaction. The presence of substantial genetic diversity across all attributes suggests that most salt-tolerant genotypes identified in this study can be incorporated into future breeding programs (Table 3). Similar T × G interactions under salinity have been reported in barley (Thabet et al. 2021), cotton (Gul et al. 2025), and sorghum (Rajabi Dehnavi et al. 2024), as well as drought-stricken barley (Badr et al. 2025). Further research is required to determine whether integrating different priming approaches, such as nano-priming, can enhance stress tolerance. Exploring combined strategies may provide synergistic effects, improving plant resilience under salinity and other abiotic stresses.

Salinity increased most biochemical traits except Pro and K⁺/Na⁺ ratio, which declined in unprimed and hydro-primed seeds. Nano-priming enhanced all traits under salinity, showing strong protective effects, especially in the tolerant genotypes. Tolerant genotypes maintained higher osmolytes, antioxidant activity, and ion balance, though they showed reduced P and lower POD under (UP) and TAC under (H). Sensitive genotypes generally increased most traits under salinity, but lost Pro and K⁺/Na⁺ stability (Supplementary Fig. S1). Pro dropped in all genotypes under unprimed and hydro-primed salinity, yet nano-priming raised protein in sensitive genotypes above the control. Our findings are consistent with those of Ashwini et al. (2024), who reported that priming with zinc oxide nanoparticles increased chlorophyll, soluble protein, total soluble sugar, and free amino acids in leaves of groundnut. Similarly, Karami and Sepehri (2018) reported that combined application of nano TiO₂ (2000 mg kg⁻^1^) and sodium nitroprusside under 100–200 mM NaCl increased total soluble protein by 47% and 43.6%, respectively. These treatments appear to protect soluble proteins by activating antioxidant enzymes, improving nitrate absorption, and accelerating inorganic nitrogen conversion, thereby promoting nitrogen metabolism. Sodium nitroprusside and nano TiO₂ appear to protect total soluble protein by stimulating antioxidant enzyme activity. In addition, nano TiO₂ enhances nitrate absorption and accelerates the conversion of inorganic nitrogen, thereby promoting nitrogen metabolism. These mechanisms suggest that nano-priming can strengthen protein stability and the protein synthesis machinery, ultimately improving tolerance to salinity. These findings align with those of Ghani et al. (2022), who demonstrated that zinc oxide nanoparticles increased the protein content of cucumber seedlings by mitigating the adverse effects of drought stress. Likewise, our results are consistent with those of El-Shazoly et al. (2025), who demonstrated that drought stress, particularly under severe conditions (60% FC), enhanced the accumulation of soluble proteins and amino acids in both shoots and roots of wheat. Consistent with this, salt-tolerant varieties of barley and rice have been reported to maintain higher levels of soluble proteins under stress, reinforcing the role of protein stability in salinity adaptation (Hurkman et al. 1989; Lutts et al. 1996). Similar trends were reported by Sen and Alikamanoglu (2011), who reported that certain salt-tolerant genotypes exhibited increased protein content at specific salt concentrations. This finding reinforces the idea that protein accumulation is a key adaptive mechanism contributing to salinity tolerance.

In line with these results, Li et al. (2008) observed that sodium nitroprusside enhances total soluble protein in stressed plants by stimulating protein synthesis, preventing protein denaturation, and improving carbon–nitrogen metabolism.

Our findings differ from those reported by Akhzari and Pessarakli (2016), who observed reduced protein accumulation in several plant species subjected to drought stress compared with control conditions. In tolerant genotypes, the K⁺/Na⁺ ratio consistently declined under all treatments during salt stress, reflecting a pronounced disruption of ionic balance. Sensitive genotypes exhibited a similar decline under unprimed conditions, while hydro-priming maintained the ratio at levels comparable to the control. This reduction indicates impaired ion homeostasis, driven by excessive Na^⁺^ accumulation and reduced K^⁺^ uptake, a well-documented physiological response in plants exposed to saline environments. Notably, sensitive genotypes subjected to nano-priming displayed a modest increase in the K⁺/Na⁺ ratio relative to the control, suggesting that nanoparticles enhanced ion selectivity and transport efficiency. These results align with previous studies demonstrating that nanomaterials can improve nutrient acquisition under salt stress, thereby reducing Na⁺ influx and promoting K^⁺^ retention to sustain resistance and productivity in saline soils (Hussein and Abou-Baker 2018). These findings further highlight the beneficial role of nano-priming in sustaining ion homeostasis and underscore the importance of K⁺/Na⁺ regulation as a central determinant of salt tolerance. Across all treatments, the Na⁺/K⁺ ratio increased in tolerant genotypes under salinity, indicating that, despite their relative resilience, these lines were unable to fully prevent Na⁺ accumulation in relation to K⁺ uptake. This outcome reflects the inherent challenge of maintaining ionic balance under saline conditions and suggests that tolerance in barley is not based on complete exclusion of Na⁺, but rather on mechanisms such as partial restriction of Na⁺ influx, enhanced K⁺ retention, and compensatory antioxidant activity.

In agreement with these findings, Sabagh et al. (2019) reported that, relative to controls, both wild and cultivated barley genotypes exhibited elevated Na⁺ concentrations and Na⁺/K⁺ ratios in roots, stems, and leaves under salinity alone and combined drought-salinity stress. Sensitive genotypes in the present study showed a similar trend: under unprimed and hydro-primed conditions, the Na⁺/K⁺ ratio increased markedly. However, following nano-priming, the ratio decreased significantly compared with the control, indicating that ZnO nanoparticles enhanced ionic regulation by reducing Na⁺ accumulation or promoting K⁺ retention. This improvement in ion selectivity highlights the capacity of nanomaterials to mitigate salinity-induced ionic imbalance. As emphasized by Munns and Tester (2008), maintaining a high cytoplasmic K⁺/Na⁺ balance is essential for salt tolerance and is achieved through a combination of morphological, physiological, and biochemical adaptations.

Phosphorus (P) is a vital macronutrient involved in energy transfer, nucleic acid synthesis, and membrane stability. In the present study, P content increased under salinity when seeds were primed, particularly with nanoparticles, whereas it declined sharply in tolerant genotypes exposed to salt stress without priming. The enhancement of P accumulation under nano-priming suggests that nanoparticles facilitated nutrient uptake and improved metabolic efficiency under stress conditions. This observation is consistent with the findings of Bachai and Jamil (2025), who reported that zinc foliar application in barley enhanced nitrogen and phosphorus concentrations in grains through multiple physiological pathways. Together, these results highlight the role of nano-based interventions in sustaining phosphorus availability and emphasize its importance in maintaining cellular function and stress resilience under saline environments.

Zinc is a cofactor for numerous enzymes involved in carbohydrate synthesis and degradation, thereby influencing energy metabolism and phosphorus utilization. Consequently, zinc plays a pivotal role in regulating phosphorus metabolism in plants (Niu et al. 2021). In the present study, the superior growth performance observed in tolerant genotypes compared with sensitive ones may be partly attributed to their ability to maintain higher phosphorus levels during nano-priming. This enhanced P availability likely supported key metabolic and physiological processes under salinity stress, contributing to improved resilience and growth stability.

In the present study, all treatments (unprimed, hydro-primed, and nano-primed) consistently resulted in elevated levels of carbohydrates, proline, Na⁺, K⁺, catalase (CAT) activity, and glutathione S-transferase (GST) activity under salinity compared with the control in both tolerant and sensitive genotypes. This universal increase reflects barley’s adaptive response to salt stress. Similar accumulation of carbohydrates under NaCl salinity has been reported in earlier studies, where starch and soluble sugars were found to build up due to impaired utilization (Greenway and Munns 1980; Rathert 1984; Munns and Termaat 1986). Comparable findings have been documented in other species, including *Sorghum bicolor* (Faheed et al. 2005), *Oryza sativa* (Amirjani 2010), and *Sesbania grandiflora* (Dhanapackiam and Ilyas 2010), where polyamine and carbohydrate accumulation were identified as effective mechanisms for salinity tolerance.

In our study, proline levels increased markedly under salt stress, particularly with nano-priming, while CAT, GST, and their associated gene expression were also enhanced. These results demonstrate that ZnO nanoparticles reinforce stress response pathways, supporting osmotic adjustment and sustaining turgor pressure, which are essential for salinity tolerance (Rady et al. 2019).

Our findings are consistent with Babaei et al. (2017), who showed that micronutrient applications such as iron and zinc increased proline, chlorophyll, soluble sugars, antioxidant enzyme activity, and yield in wheat under salinity. Proline is a well-known osmolyte that mediates osmotic adjustment and protects subcellular structures, with its accumulation positively correlated with stress tolerance (Yang et al. 2003). Osmolyte accumulation has been proposed as a selection criterion for salt tolerance (Ashraf 2004; Lee et al. 2001). Hydro-priming, however, resulted in a significant reduction in peroxidase (POD) activity in tolerant genotypes, suggesting that hydro-priming alone was insufficient to provide effective antioxidant defense. In contrast, POD activity increased under salinity without priming and with nano-priming. The activation of antioxidant and detoxification mechanisms, particularly CAT, POD, and GST, confirms their critical role in scavenging reactive oxygen species (ROS) and maintaining redox equilibrium under stress. The increases in CAT and POD observed here align with previous reports in barley primed with nano iron and zinc (Dadashzadeh et al. 2018) and with Mittova et al. (2003), who demonstrated enhanced antioxidant enzyme activity under salinity. Similar increases in SOD, POD, and CAT have been documented with TiO₂ and Ag nanoparticles in *Zea mays* and *Solanum lycopersicum* (Shah et al. 2021; Sonawane et al. 2021), as well as in *Psophocarpus tetragonolobus* primed with Ag nanoparticles (Kumar et al. 2020). These steady increases across both tolerant and sensitive genotypes suggest that antioxidant activation is a fundamental response to salinity stress. By rapidly scavenging ROS, plants maintain ROS concentrations below harmful thresholds (Hashem et al. 2015).

Numerous studies have demonstrated that ZnO nanoparticles (ZnO-NPs) enhance the antioxidant defense systems of plants through diverse mechanisms. They stimulate the activity of key antioxidant enzymes, mitigate the accumulation of reactive oxygen species (ROS), and promote redox balance, thereby strengthening plant tolerance to environmental stress (Ochoa-Chaparro et al. 2026).

For instance, Gupta et al. (2024) found that ZnO-NPs applied under saline conditions significantly enhanced antioxidant enzyme activity (SOD, CAT), elevated proline accumulation, and improved ionic homeostasis, collectively contributing to greater stress resilience.

Beyond their direct enzymatic effects, ZnO-NPs also appear to influence redox signaling pathways. Singh et al. (2025) demonstrated that foliar application of ZnO-NPs elevated non-enzymatic antioxidants such as glutathione (GSH) and ascorbate (AsA) in stressed plants, reinforcing ROS-scavenging capacity and redox stability. These molecules not only detoxify peroxides but also act as signaling intermediates, enabling plants to fine-tune gene expression in response to oxidative stress. Further evidence from Ali et al. (2025) revealed that ZnO-NPs regulate glutathione redox status by increasing GSH levels and improving the GSH/GSSG ratio in *Brassica napus*. This shift in redox potential enhances cellular reducing power, safeguards protein thiol groups, and prevents irreversible oxidative damage.

Collectively, these findings highlight ZnO-NPs as not merely passive antioxidants but active modulators of cellular redox networks. Their role extends to transcriptional regulation, stress signaling, and the reinforcement of physiological resilience under diverse abiotic stress conditions (Ochoa-Chaparro et al. 2026).

The contrasting responses between tolerant and sensitive genotypes indicate that tolerance is associated with balanced regulation of antioxidant enzymes and osmotic adjustment, rather than maximal induction of POD. Tolerant genotypes maintained a more efficient antioxidant and osmolyte-based strategy, while sensitive genotypes exhibited less stable ion balance, particularly in terms of the K⁺/Na⁺ ratio. The superior performance of nano-priming in enhancing biochemical traits underscores its role in reinforcing stress tolerance pathways, consistent with earlier reports on nanoparticle-mediated priming in cereals. Under salinity and nano-priming, the pronounced biochemical differences between tolerant and sensitive genotypes suggest that these responses are underpinned by molecular regulation. Sensitive genotypes were unable to sustain protective adaptations, whereas tolerant genotypes maintained improved Na⁺/K⁺ balance, greater osmolyte accumulation, and stronger antioxidant activity. To validate these biochemical findings, we examined the expression of key genes involved in ion transport and signaling (*SOS1*, *SOS3*, *NHX1*, *NHX2*, *NHX3*) and antioxidant defense (*APX*, *CAT1*, *GR*, *GPX*, *SODA*), confirming that these adaptive features were supported at the transcriptional level.

### Gene expression

Five barley genotypes, three tolerant (BCC1398, BCC1416, and HOR11370) and two sensitive (BCC1505 and BCC532), were evaluated for the expression of ten key genes involved in ion transport and antioxidant defense (*NHX1*, *NHX2*, *NHX3*, *SOS1*, *SOS3*, *CAT1*, *APX*, *SODA, GPX*, and *GR*). Gene expression patterns varied markedly across genotypes and treatments, highlighting genotype-specific regulatory mechanisms and genotype-treatment interaction.

Under salinity relative to the control, the tolerant genotypes differentially expressed the genes; most genes were upregulated in the tolerant genotypes HOR11370 and BCC1398, whereas all ten genes were downregulated in the tolerant genotype BCC1416. A similar pattern has been observed in the sensitive genotypes, BCC1505 showed upregulation of all genes except *GR* and *SOS1*, while BCC532 exhibited downregulation of most genes, except *APX*, *SODA*, and *SOS1*.

When comparing salinity with nano-priming relative to salinity alone, tolerant genotypes again displayed differential responses. HOR11370 and BCC1416 showed upregulation of most genes, whereas BCC1398 exhibited downregulation of all genes except *CAT1*. In sensitive genotypes, BCC1505 showed downregulation of all genes except *CAT1* and *GR*, while BCC532 demonstrated upregulation of most genes, except for *NHX2*, *SODA*, and *SOS1*.

These results confirm substantial genotype-specific variation in gene expression both within tolerant and sensitive groups and between them. Variations in *NHX*, *SOS*, and antioxidant-related genes underscore distinct tolerance mechanisms: tolerant genotypes tend to activate ion transport and antioxidant pathways under stress, while sensitive genotypes display less consistent or compensatory responses.

Our findings align with those of Nakayama et al. (2022), who demonstrated that in salt-tolerant common wheat, the expression of salt-responsive genes followed genotype-specific patterns, with the Na⁺/H⁺ antiporter gene being significantly upregulated in some tolerant lines but downregulated in others. Under salinity relative to control, *NHX3* showed exceptionally high induction in the tolerant genotype BCC1398 (208-fold), underscoring its role in vacuolar Na⁺ sequestration and ionic balance. This finding is consistent with reports that *NHX* genes are central to osmotic regulation, stomatal function, and reproductive development under stress (Bassil et al. 2019). The differential regulation of *NHX* isoforms across tolerant and sensitive genotypes in our study reflects the complexity of adaptive strategies in barley.

Plants typically rely on coordinated ion transport, compartmentalization, and distribution during salinity stress, mediated by gene families such as *HvSOS*, *HvHKT*, and *HvNHX*. Among these, the Na⁺/H⁺ antiporter (*NHX*) is particularly critical.

*NHX* proteins are generally classified into vacuolar isoforms* (AtNHX1-4)* and endosomal isoforms* (AtNHX5-6)* in Arabidopsis (Barragán et al. 2012). In barley, four isoforms (*HvNHX1-4*) have been identified, all primarily associated with vacuolar membranes (Jabeen et al. 2022). The strong induction of *NHX3* in tolerant genotypes in our study suggests that vacuolar sequestration of Na⁺ is a key tolerance mechanism; this may explain the high levels of Na^+^ in the tolerant genotypes. Meanwhile, this set of genes showed weaker or inconsistent responses in sensitive genotypes, which may explain their reduced resilience.

Complementing *NHX*‑mediated compartmentalization, the Salt‑Overly‑Sensitive (SOS) pathway safeguards tissues against excessive Na⁺ accumulation (Ji et al. 2013). This pathway involves three core genes, *SOS1*, *SOS2*, and *SOS3,* whose expression is activated under salt stress (Shi et al. 2002). In our study, the relative expression of *SOS1* under salinity with nano-priming (compared to salinity alone) showed a positive correlation with Na^⁺^ concentration, while *SOS3* exhibited a modest correlation with Na^⁺^ levels, consistent with its role in mediating Na^⁺^ efflux and xylem loading. Previous work confirmed that *SOS1* is essential for xylem Na⁺ transport and long‑distance ion regulation (Olías et al. 2009). This suggests that both genes contribute to regulating Na^⁺^ exclusion and maintaining tissue ion homeostasis, particularly under nanoparticle-mediated priming. The genotype-specific responses observed under salinity relative to control, such as the strong induction of *SOS1* in tolerant genotypes (BCC1398 and HOR11370) and the moderate upregulation in sensitive genotype BCC532, highlight the important role of the *SOS* pathway in balancing Na⁺ transport under stress.

The clustering of *NHX3* with the K⁺/Na⁺ ratio in our heatmap analysis further supports the functional interplay between vacuolar sequestration and cytosolic Na⁺ exclusion, both of which are reinforced by nano-priming. Together, these results demonstrate that nano-priming enhances the transcriptional regulation of *SOS* and *NHX* genes, thereby strengthening ionic balance and stress resilience. The genotype-specific variation observed in our study underscores the importance of integrating molecular and biochemical traits when selecting barley lines for breeding programs aimed at salinity tolerance.

The expression profiles of *GR* and *APX* genes showed pronounced changes under salinity and ZnO nanoparticle treatments, particularly in the tolerant genotypes BCC1416 and HOR11370, as well as in the sensitive genotype BCC532. This pattern suggests transcriptional regulation of the glutathione-ascorbate antioxidant pathway, even though the enzymatic activities of this cycle were not directly measured in the present investigation. Our findings are consistent with those of Adhikari et al. (2020), who reported that Zea mays seedlings treated with nano‑ZnO exhibited decreased glutathione levels but increased glutathione reductase activity. Such regulation of *GR* and *APX* expression implies that the glutathione-ascorbate cycle played an active role in maintaining redox balance under stress. In plants, this cycle is fundamental for redox homeostasis: hydrogen peroxide is reduced by ascorbate via *APX*, dehydroascorbate is subsequently reduced by glutathione, and oxidized glutathione is restored by GR. These processes occur in multiple cellular compartments, including the cytosol, mitochondria, plastids, and peroxisomes (Bartoli et al. 2018). The activation of the antioxidant defense system in barley exposed to salinity and nano‑priming was further supported by increased activity of GST, POD, and CAT. This agrees with previous reports (Barros et al. 2019; Jahani et al. 2020; Lu et al. 2020; Sharma et al. 2019; Wahid et al. 2020), which demonstrated that metal oxide nanoparticles significantly modulate antioxidant enzyme activities and alter both low‑ and high‑molecular‑weight antioxidant compounds. Transcriptional analysis of *CAT1*, encoding catalase isozymes, revealed consistent upregulation in all genotypes under salinity with nano‑priming compared to salinity alone. These results parallel those of Azarin et al. (2022), who observed increased *CAT1* expression in leaf tissue at moderate nano‑ZnO concentrations (300 mg/L), but reduced expression at higher levels (2000 mg/L), highlighting the dose‑dependent nature of nanoparticle effects.

The influence of ZnO nanoparticles (ZnO-NPs) in plants extends beyond immediate physiological adjustments, driving transcriptional reprogramming that regulates genes associated with antioxidant defense, ion transport, photosynthesis, hormonal signaling, and metal detoxification. This molecular flexibility is fundamental to plant adaptation under diverse abiotic stress conditions (Ochoa-Chaparro et al. 2026). A consistent outcome of ZnO-NP exposure is the induction of genes encoding antioxidant enzymes. For instance, in tomato plants subjected to salinity stress, ZnO-NPs activated the expression of *SOD*, *CAT*, and *APX*, leading to a marked reduction in ROS accumulation and lipid peroxidation (Ahmed et al. 2025). Integrated transcriptomic and metabolomic analyses further revealed that ZnO-NPs reshape antioxidant-related gene expression and modulate secondary metabolic pathways, particularly those linked to phenylpropanoid and flavonoid biosynthesis (Sun et al. 2020). Under saline conditions, ZnO-NPs also regulate genes involved in ion transport and cellular ionic homeostasis. Notably, the overexpression of *NHX* and *HKT1*, which facilitate Na⁺ exclusion and K⁺ retention, along with *SOS1*, which mediates Na⁺ efflux at the plasma membrane, has been documented. These transcriptional changes enable plants to sustain functional ionic balance even under high salinity stress (Singh et al. 2024).

Taken together, the antioxidant system in barley responded effectively at both transcriptional and enzymatic levels, as evidenced by enhanced CAT, POD, and GST activities that aligned with the upregulation of their respective genes. By integrating molecular and biochemical data, this study establishes a clear link between transcriptional regulation and physiological performance, providing deeper insight into how nano‑priming strengthens barley’s capacity to withstand salinity stress.

## Conclusion

This study demonstrates that ZnO nanoparticle seed priming significantly enhances barley tolerance to salinity stress through coordinated biochemical, physiological, and molecular mechanisms. Nano‑priming improved Osmo protectant accumulation (proline, carbohydrates), strengthened antioxidant defenses (CAT, POD, GST, TAC), and promoted a favorable ionic balance through increased K^⁺^ retention and reduced Na^⁺^ uptake. At the transcriptional level, tolerant genotypes exhibited strong induction of *NHX* and *SOS* genes, particularly *HvNHX3* and *HvSOS1*, while antioxidant genes such as *CAT1*, *APX*, and *GR* were upregulated in both tolerant and sensitive genotypes under nano‑priming. These molecular changes were tightly correlated with enhanced enzyme activities, confirming that transcriptional regulation translated into physiological resilience. Multivariate analysis (PCA and clustering) of biochemical traits and gene expression revealed that nano‑priming not only reinforced stress‑responsive pathways but also reduced genotype‑specific differences, enabling partial adaptation in sensitive lines. Collectively, these findings highlight that salinity tolerance in barley is governed by the synergistic regulation of ion transporters and antioxidant systems, and that ZnO nanoparticle priming strengthens this coordination. By establishing clear links between molecular regulation and physiological performance, this work provides valuable insights for breeding and agronomic strategies aimed at improving barley productivity in salt‑affected soils. Nano‑priming emerges as a promising, sustainable approach to enhance crop resilience, offering practical applications for agriculture in saline environments.

## Supplementary Information

Below is the link to the electronic supplementary material.

Supplementary file1 (PPTX 1259 KB) Fig. S1 Histogram of the difference in the biochemical traits under control and salinity stress between the most tolerant genotypes (A) and sensitive (B). Where, Protein (Pro), Carbohydrates (C), Sodium (Na), Potassium (K), Potassium/sodium ratio (K/Na), Sodium/potassium ratio (Na/K), Phosphorus (P), Peroxidase (POD), Glutathione-S-Transferase (GST), Catalase (CAT), and Total antioxidant capacity (TAC)Fig. S2 The fold change of each genotype under salinity compared to control for all traits (Protein content, Carbohydrate content, Proline content, Sodium ion, Potassium ion, K/Na ratio, Na/K ratio, Phosphorus content, Peroxidase activity, Glutathione_S_Transferase activity, Catalase activity, and Total Antioxidant Capacity) under all treatments (unprimed, hydro-priming, and nano-priming conditions)Fig. S3 Principal component analysis for 10 contrasting barley genotypes under control and salinity for all treatments; a) under unprimed conditions (UP), b) under hydro-priming conditions (H), and c) under nano-priming conditions (N). Where, Pro, C, Pr, Na, K, K/Na, Na/K, P, POD, GST, CAT, and TAC refer to protein, carbohydrate, proline, sodium, potassium, potassium/sodium ratio, sodium/ potassium ratio, phosphorus, peroxidase, glutathione-s-transferase, catalase, and total antioxidant capacityFig. S4 Clustering heatmap for 10 contrasting barley genotypes under control and salinity for all treatments a) control under unprimed conditions (C_UP), b) salinity under unprimed conditions (S_UP), c) control under hydro-priming conditions (C_H), d) salinity under hydro-priming conditions (S_H), e) control under nano-priming conditions (C_N), and f) salinity under nano-priming conditions (S_N). Where, Pro, C, Pr, Na, K, K/Na, Na/K, P, POD, GST, CAT, and TAC refer to protein, carbohydrate, proline, sodium, potassium, potassium/sodium ratio, sodium/ potassium ratio, phosphorus, peroxidase, glutathione-s-transferase, catalase, and total antioxidant capacity. The x-axis represents the genotypes of barleyFig. S5 Heatmap for log2FC of all genes in five contrasting barley genotypes: BCC1398, BCC1416, HOR11370 “tolerant”, BCC1505, and BCC532 “sensitive”. Where S_UP and S_N stand for Salt vs control and Salt + nano vs Salt

Supplementary file2 (XLSX 16 KB) Table S1 Minimum, maximum, and mean for all traits scored on ten contrasting barley genotypes under control and salt stress (200 mM NaCl) conditions for unprimed seeds.Table S2 Minimum, maximum, and mean for all traits scored on ten contrasting barley genotypes under control and salt stress (200 mM NaCl) conditions for hydro-primed seeds.Table S3 Minimum, maximum, and mean for all traits scored on ten contrasting barley genotypes under control and salt stress (200 mM NaCl) conditions for nano-primed seeds.Table S4 Analysis of variance (ANOVA) for all genes (HvAPX, HvCAT1, HvGPX, HvGR, HvNHX1, HvNHX2, HvNHX3, HvSODA, HvSOS1, and HvSOS3) under all treatments (control, salinity, and salinity with nano-priming)

## Data Availability

All data generated or analyzed during this study are included in this article and its supplementary information files. All materials are available through the corresponding authors upon reasonable request.

## References

[CR1] Abdelhamid MT, Rady MM, Osman AS, Abdalla MA (2013) Exogenous application of proline alleviates salt-induced oxidative stress in Phaseolus vulgaris L. plants. J Horticult Sci Biotechnol 88(4):439–446. 10.1080/14620316.2013.11512989

[CR2] Abozaid WW, Thabet SG, Karam MA, Moursi YS (2025) GWAS-based identification of multi-trait genetic loci conferring salinity tolerance in barley under hydro- and nanoparticle-priming conditions. BMC Plant Biol. 10.1186/s12870-025-07898-5 41437322 10.1186/s12870-025-07898-5 PMC12849565

[CR3] Adem GD, Roy SJ, Zhou M, Bowman JP, Shabala S (2014) Evaluating contribution of ionic, osmotic and oxidative stress components towards salinity tolerance in barley. BMC Plant Biol 14(1):113. 10.1186/1471-2229-14-113 24774965 10.1186/1471-2229-14-113 PMC4021550

[CR4] Adhikari S, Adhikari A, Ghosh S, Roy D, Azahar I, Basuli D, Hossain Z (2020) Assessment of ZnO-NPs toxicity in maize: an integrative microRNAomic approach. Chemosphere 249:126197. 10.1016/j.chemosphere.2020.126197 32087455 10.1016/j.chemosphere.2020.126197

[CR5] Adil M et al (2022) Zinc oxide nanoparticles improved chlorophyll contents, physical parameters, and wheat yieldunder salt stress. Front Plant Sci 13:932861 35991444 10.3389/fpls.2022.932861 PMC9382196

[CR6] Aebi H (1984) Catalase in vivo. *Methods in **Enzymol*. Oxygen Radical Biol Syst 105:121–126 10.1016/s0076-6879(84)05016-3 6727660

[CR7] Ahmed M, Tóth Z, Rizk R, Abdul-Hamid D, Decsi K (2025) Investigation of antioxidative enzymes and transcriptomic analysis in response to foliar application of zinc oxide nanoparticles and salinity stress in *Solanum lycopersicum*. Agronomy 15(7):1715

[CR8] Akhzari D, Pessarakli M (2016) Effect of drought stress on total protein, essential oil content, and physiological traits of *Levisticum officinale* Koch. J Plant Nutr 39(10):1365–1371. 10.1080/01904167.2015.1109125

[CR9] Alhammad BA, Ahmad A, Seleiman MF, Tola E (2023) Seed priming with nanoparticles and 24-epibrassinolide improved seed germination and enzymatic performance of Zea mays L. in salt-stressed soil. Plants 12(4):690. 10.3390/plants12040690 36840038 10.3390/plants12040690 PMC9963209

[CR10] Ali B et al (2022) RETRACTED: Mitigation of salinity stress in barley genotypes with variable salt tolerance byapplication of zinc oxide nanoparticles. Front Plant Sci 13:973782 36072329 10.3389/fpls.2022.973782 PMC9441957

[CR11] Ali S, Ali B, Sajid IA, Ahmad S, Yousaf MA, Ulhassan Z, Zhang K, Ali S, Zhou W, Mao B (2025) Synergistic effects of exogenous melatonin and zinc oxide nanoparticles in alleviating cobalt stress in *Brassica napus*: insights from stress-related markers and antioxidant machinery. Environ Sci Nano 12(1):368–387

[CR12] Alsamadany H, Alharby HF, Al-Zahrani HS, Kuşvuran A, Kuşvuran S, Rady MM (2023) Selenium fortification stimulates antioxidant-and enzyme gene expression-related defense mechanisms in response to saline stress in *Cucurbita pepo*. Sci Hortic 312:111886. 10.1016/j.scienta.2023.111886

[CR13] Amirjani MR (2010) Effect of NaCl on some physiological parameters of Rice. Ejbs 3(June):6–16

[CR14] Arif Y, Singh P, Siddiqui H, Bajguz A, Hayat S (2020) Salinity induced physiological and biochemical changes in plants: an omic approach towards salt stress tolerance. Plant Physiol Biochem 156:64–77. 10.1016/j.plaphy.2020.08.042 32906023 10.1016/j.plaphy.2020.08.042

[CR15] Ashraf M (2004) Some important physiological selection criteria for salt tolerance in plants. Flora Jena 199(5):361–376. 10.1078/0367-2530-00165

[CR16] Ashwini MN, Gajera HP, Hirpara DG, Savaliya DD, Kandoliya UK (2024) Comparative impact of seed priming with zinc oxide nanoparticles and zinc sulphate on biocompatibility, zinc uptake, germination, seedling vitality, and antioxidant modulation in groundnut. J Nanopart Res 26(10):235. 10.1007/s11051-024-06141-w

[CR17] Azarin K, Usatov A, Minkina T, Plotnikov A, Kasyanova A, Fedorenko A, Duplii N, Vechkanov E, Rajput VD, Mandzhieva S (2022) Effects of ZnO nanoparticles and its bulk form on growth, antioxidant defense system and expression of oxidative stress related genes in *Hordeum vulgare* L. Chemosphere 287:132167. 10.1016/j.chemosphere.2021.132167 34509010 10.1016/j.chemosphere.2021.132167

[CR18] Babaei K, Seyed Sharifi R, Pirzad A, Khalilzadeh R (2017) Effects of bio fertilizer and nano Zn-Fe oxide on physiological traits, antioxidant enzymes activity and yield of wheat (Triticum aestivum L.) under salinity stress. Journal of Plant Interactions 12(1):381–389. 10.1080/17429145.2017.1371798

[CR19] Bachai HS, Jamil DAK (2025) Effect of Bulk Zinc and Nano Zinc on the Mineral Elements Content of Barley Plant under Salt Stress. J Nanostruct 15(3):1168–1177

[CR20] Badr A, El-Shazly HH, Mahdy M, Schierenbeck M, Helmi RY, Börner A, Youssef HM (2025) GWAS identifies novel loci linked to seedling growth traits in highly diverse barley population under drought stress. Sci Rep 15(1):10085. 10.1038/s41598-025-94175-y 40128265 10.1038/s41598-025-94175-y PMC11933270

[CR21] Balasubramaniam T, Shen G, Esmaeili N, Zhang H (2023) Plants’ response mechanisms to salinity stress. Plants Basel 12(12):2253. 10.3390/plants12122253 37375879 10.3390/plants12122253 PMC10300796

[CR22] Barragán V, Leidi EO, Andrés Z, Rubio L, De Luca A, Fernandez JA, Cubero B, Pardo JM (2012) Ion exchangers NHX1 and NHX2 mediate active potassium uptake into vacuoles to regulate cell turgor and stomatal function in *Arabidopsis*. Plant Cell 24(3):1127–1142. 10.1105/tpc.111.095273 22438021 10.1105/tpc.111.095273 PMC3336136

[CR23] Barros D, Pradhan A, Mendes VM, Manadas B, Santos PM, Pascoal C, Cássio F (2019) Proteomics and antioxidant enzymes reveal different mechanisms of toxicity induced by ionic and nanoparticulate silver in bacteria. Environ Sci Nano 6(4):1207–1218

[CR24] Bartoli CG, Buet A, GergoffGrozeff G, Galatro A, Simontacchi M (2018) Ascorbate-glutathione cycle and abiotic stress tolerance in plants. Ascorbic acid in plant growth, development and stress tolerance. Springer, pp 177–200

[CR25] Bassil E, Zhang S, Gong H, Tajima H, Blumwald E (2019) Cation specificity of vacuolar NHX-type cation/H+ antiporters. Plant Physiol 179(2):616–629. 10.1104/pp.18.01103 30498025 10.1104/pp.18.01103 PMC6426403

[CR26] Bates LS, Waldren RP, Teare ID (1973) Rapid determination of free proline for water-stress studies. Plant Soil 39(1):205–207. 10.1007/BF00018060

[CR27] Bradford MM (1976) A rapid and sensitive method for the quantitation of microgram quantities of protein utilizing the principle of protein-dye binding. Anal Biochem 72(1–2):248–254. 10.1016/0003-2697(76)90527-3 942051 10.1016/0003-2697(76)90527-3

[CR28] Cembrowska-Lech D, Rybak K (2023) Nanopriming of barley seeds—a shotgun approach to improve germination under salt stress conditions by regulating of reactive oxygen species. Plants (Basel) 12(2):405. 10.3390/plants12020405 36679118 10.3390/plants12020405 PMC9864488

[CR29] Chapman HD, Pratt PF (1962) Methods of analysis for soils, plants and waters. Soil Sci 93(1):68

[CR30] Chen Z, Cuin TA, Zhou M, Twomey A, Naidu BP, Shabala S (2007) Compatible solute accumulation and stress-mitigating effects in barley genotypes contrasting in their salt tolerance. J Exp Bot 58(15–16):4245–4255 18182428 10.1093/jxb/erm284

[CR31] Dadashzadeh S, Shariff RS, Farzaneh S (2018) Physiological and biochemical responses of barley to application of bio-fertilizers and nano iron oxide under salinity stress in greenhouse. Bangladesh J Botany 47(4):863–875. 10.3329/bjb.v47i4.47364

[CR32] Dawson IK, Russell J, Powell W, Steffenson B, Thomas WTB, Waugh R (2015) Barley: a translational model for adaptation to climate change. New Phytol 206(3):913–931. 10.1111/nph.13266 25605349 10.1111/nph.13266

[CR33] Debez A, Ben Slimen ID, Bousselmi S, Atia A, Farhat N, El Kahoui S, Abdelly C (2020) Comparative analysis of salt impact on sea barley from semi-arid habitats in Tunisia and cultivated barley with special emphasis on reserve mobilization and stress recovery aptitude. Plant Biosyst 154(4):544–552. 10.1080/11263504.2019.1651777

[CR34] Dhanapackiam S, Ilyas M (2010) Effect of salinity on chlorophyll and carbohydrate contents of *Sesbania grandiflora* seedlings. Indian J Sci Technol 3(1):64–66

[CR35] Donia DT, Carbone M (2023) Seed priming with zinc oxide nanoparticles to enhance crop tolerance to environmental stresses. Int J Mole Science 24(24):17612 10.3390/ijms242417612 PMC10744145 38139445

[CR36] DuBois M, Gilles KA, Hamilton JK, Rebers PA, Smith F (1956) Colorimetric method for determination of sugars and related substances. Anal Chem 28(3):350–356. 10.1021/ac60111a017

[CR37] El-Badri AMA et al (2021) Modulation of salinity impact on early seedling stage via nano-priming application of zinc oxide on rapeseed (*Brassica napus* L.). Plant Physiol Biochem 166:376–392 34153882 10.1016/j.plaphy.2021.05.040

[CR38] El-Shazoly RM, Othman AA, Zaheer MS, Al-Hossainy AF, Abdel-Wahab DA (2025) Zinc oxide seed priming enhances drought tolerance in wheat seedlings by improving antioxidant activity and osmoprotection. Sci Rep 15(1):3863. 10.1038/s41598-025-86824-z 39890839 10.1038/s41598-025-86824-z PMC11785979

[CR39] Faheed FA, Hassanein AM, Azooz MM (2005) Gradual increase in nacl concentration overcomes inhibition of seed germination due to salinity stress in Sorghum Bicolor (L.). Acta Agron Hung 53(2):229–239. 10.1556/AAgr.53.2005.2.11

[CR40] Faizan M et al (2021) Zinc oxide nanoparticles (ZnO-NPs) induce salt tolerance by improving the antioxidantsystem and photosynthetic machinery in tomato. Plant Physiol Biochem 161:122–130 33581620 10.1016/j.plaphy.2021.02.002

[CR41] Fatima F, Hashim A, Anees S (2021) Efficacy of nanoparticles as nanofertilizer production: a review. Environ Sci Pollut Res 28(2):1292–1303. 10.1007/s11356-020-11218-9 10.1007/s11356-020-11218-9 33070292

[CR42] Flowers TJ, Hajibagheri MA (2001) Salinity tolerance in *Hordeum vulgare*: ion concentrations in root cells of cultivars differing in salt tolerance. Plant Soil 231(1):1–9. 10.1023/A:1010372213938

[CR43] Ghani MI, Saleem S, Rather SA, Rehmani MS, Alamri S, Rajput VD, Kalaji HM, Saleem N, Sial TA, Liu M (2022) Foliar application of zinc oxide nanoparticles: an effective strategy to mitigate drought stress in cucumber seedling by modulating antioxidant defense system and osmolytes accumulation. Chemosphere 289:133202. 10.1016/j.chemosphere.2021.133202 34890613 10.1016/j.chemosphere.2021.133202

[CR44] Greenway H, Munns R (1980) Mechanisms of salt tolerance in nonhalophytes. Annu Rev Plant Physiol 31(1):149–190

[CR45] Gul N, Khan Z, Shani MY, Hafiza BS, Saeed A, Khan AI, Shakeel A, Rahimi M (2025) Identification of salt-resilient cotton genotypes using integrated morpho-physiological and biochemical markers at the seedling stage. Sci Rep 15(1):5276. 10.1038/s41598-025-89582-0 39939688 10.1038/s41598-025-89582-0 PMC11821876

[CR46] Gupta A, Bharati R, Kubes J, Popelkova D, Praus L, Yang X, Severova L, Skalicky M, Brestic M (2024) Zinc oxide nanoparticles application alleviates salinity stress by modulating plant growth, biochemical attributes and nutrient homeostasis in *Phaseolus vulgaris* L. Front Plant Sci 15:1432258. 10.3389/fpls.2024.1432258 39297008 10.3389/fpls.2024.1432258 PMC11408239

[CR47] Habig WH, Pabst MJ, Jakoby WB (1974) Glutathione S-transferases: the first enzymatic step in mercapturic acid formation. J Biol Chem 249(22):7130–7139. 10.1016/S0021-9258(19)42083-8 4436300

[CR48] Hammerschmidt R, Nuckles EM, Kuć J (1982) Association of enhanced peroxidase activity with induced systemic resistance of cucumber to *Colletotrichum lagenarium*. Physiol Plant Pathol 20(1):73–82. 10.1016/0048-4059(82)90025-X

[CR49] Han Y, Yin S, Huang L, Wu X, Zeng J, Liu X, Qiu L, Munns R, Chen ZH, Zhang G (2018) A sodium transporter HvHKT1;1 confers salt tolerance in barley via regulating tissue and cell ion homeostasis. Plant Cell Physiol 59(10):1976–1989. 10.1093/pcp/pcy116 29917153 10.1093/pcp/pcy116

[CR50] Hashem A, Abd Allah EF, Alqarawi AA, Aldubise A, Egamberdieva D (2015) Arbuscular mycorrhizal fungi enhances salinity tolerance of *Panicum turgidum* forssk by altering photosynthetic and antioxidant pathways. J Plant Interact 10(1):230–242. 10.1080/17429145.2015.1052025

[CR51] Hurkman WJ, Fornari CS, Tanaka CK (1989) A comparison of the effect of salt on polypeptides and translatable mRNAs in roots of a salt-tolerant and a salt-sensitive cultivar of barley. Plant Physiol 90(4):1444–1456. 10.1104/pp.90.4.1444 16666950 10.1104/pp.90.4.1444 PMC1061910

[CR52] Hussein MM, Abou-Baker NH (2018) The contribution of nano-zinc to alleviate salinity stress on cotton plants. R Soc Open Sci 5(8):171809. 10.1098/rsos.171809 30224982 10.1098/rsos.171809 PMC6124091

[CR53] Jabeen Z, Irshad F, Hussain N, Han Y, Zhang G (2022) NHX-type Na+/H+ antiporter gene expression under different salt levels and allelic diversity of HvNHX in wild and cultivated barleys. Front Genet 12:809988. 10.3389/fgene.2021.809988 35273633 10.3389/fgene.2021.809988 PMC8902669

[CR54] Jahani M, Khavari-Nejad RA, Mahmoodzadeh H, Saadatmand S (2020) Effects of cobalt oxide nanoparticles (Co3O4 NPs) on ion leakage, total phenol, antioxidant enzymes activities and cobalt accumulation in Brassica napus L. Notulae Botanicae Horti Agrobotanici Cluj-Napoca 48(3):1260–1275

[CR55] Ji H, Pardo JM, Batelli G, Van Oosten MJ, Bressan RA, Li X (2013) The salt overly sensitive (SOS) pathway: established and emerging roles. Mol Plant 6(2):275–286 23355543 10.1093/mp/sst017

[CR56] Karami A, Sepehri A (2018) Nano titanium dioxide and nitric oxide alleviate salt induced changes in seedling growth, physiological and photosynthesis attributes of barley. Zemdirbyste 105(2):123–132

[CR57] Kumar VK, Muthukrishnan S, Rajalakshmi R (2020) Phytostimulatory effect of phytochemical fabricated nanosilver (AgNPs) on Psophocarpus tetragonolobus (L.) DC. seed germination: An insight from antioxidative enzyme activities and genetic similarity studies. Curr Plant Biol 23:100158

[CR58] Lee DH, Kim YS, Lee CB (2001) The inductive responses of the antioxidant enzymes by salt stress in the rice (Oryza sativa L.). J Plant Physiol 158(6):737–745. 10.1078/0176-1617-00174

[CR59] Li Q-Y, Niu H-B, Yin J, Wang M-B, Shao H-B, Deng D-Z, Chen X-X, Ren J-P, Li Y-C (2008) Protective role of exogenous nitric oxide against oxidative-stress induced by salt stress in barley (*Hordeum vulgare*). Colloids Surf B Biointerfaces 65(2):220–225. 10.1016/j.colsurfb.2008.04.007 18502620 10.1016/j.colsurfb.2008.04.007

[CR60] Lu K, Shen D, Liu X, Dong S, Jing X, Wu W, Tong Y, Gao S, Mao L (2020) Uptake of iron oxide nanoparticles inhibits the photosynthesis of the wheat after foliar exposure. Chemosphere 259:127445. 10.1016/j.chemosphere.2020.127445 32593005 10.1016/j.chemosphere.2020.127445

[CR61] Lutts S, Kinet JM, Bouharmont J (1996) Effects of salt stress on growth, mineral nutrition and proline accumulation in relation to osmotic adjustment in rice (Oryza sativa L.) cultivars differing in salinity resistance. Plant Growth Regul 19(3):207–218. 10.1007/BF00037793

[CR62] Mian A, Oomen RJFJ, Isayenkov S, Sentenac H, Maathuis FJM, Véry A (2011) Over‐expression of an Na+‐and K+‐permeable HKT transporter in barley improves salt tolerance. Plant J 68(3):468–479. 10.1111/j.1365-313X.2011.04701.x 21749504 10.1111/j.1365-313X.2011.04701.x

[CR63] Mittova V, Tal M, Volokita M, Guy M (2003) Up-regulation of the leaf mitochondrial and peroxisomal antioxidative systems in response to salt-induced oxidative stress in the wild salt-tolerant tomato species *Lycopersicon pennellii*. Plant Cell Environ 26(6):845–856. 10.1046/j.1365-3040.2003.01016.x 12803612 10.1046/j.1365-3040.2003.01016.x

[CR64] Munns R, Termaat A (1986) Whole-plant responses to salinity. Funct Plant Biol 13(1):143–160. 10.1093/aob/mcw191

[CR65] Munns R, Tester M (2008) Mechanisms of salinity tolerance. Annu Rev Plant Biol 59(1):651–681. 10.1146/annurev.arplant.59.032607.092911 18444910 10.1146/annurev.arplant.59.032607.092911

[CR66] Munns R, James RA, Xu B, Athman A, Conn SJ, Jordans C, Byrt CS, Hare RA, Tyerman SD, Tester M (2012) Wheat grain yield on saline soils is improved by an ancestral Na^+^ transporter gene. Nat Biotechnol 30(4):360–364. 10.1038/nbt.2120 22407351 10.1038/nbt.2120

[CR67] Nakayama R, Safi MT, Ahmadzai W, Sato K, Kawaura K (2022) Comparative transcriptome analysis of synthetic and common wheat in response to salt stress. Sci Rep 12(1):11534. 10.1038/s41598-022-15733-2 35798819 10.1038/s41598-022-15733-2 PMC9262916

[CR68] Ni X, Quisenberry SS, Heng-Moss T, Markwell J, Sarath G, Klucas R, Baxendale F (2001) Oxidative responses of resistant and susceptible cereal leaves to symptomatic and nonsymptomatic cereal aphid (Hemiptera: Aphididae) feeding. J Econ Entomol 94(3):743–751. 10.1603/0022-0493-94.3.743 11425032 10.1603/0022-0493-94.3.743

[CR69] Niu J, Liu C, Huang M, Liu K, Yan D (2021) Effects of foliar fertilization: a review of current status and future perspectives. J Soil Sci Plant Nutr 21(1):104–118. 10.1007/s42729-020-00346-3

[CR70] Ochoa-Chaparro EH, Castruita-Esparza LU, Sánchez E (2026) Eco-physiological and molecular roles of zinc oxide nanoparticles (ZnO-NPs) in mitigating abiotic stress: a comprehensive review. Plants 15(1):147 41515092 10.3390/plants15010147 PMC12787645

[CR71] Olías R, Eljakaoui Z, Li JUN, Morales PAZADE (2009) The plasma membrane Na + / H + antiporter SOS1 is essential for salt tolerance in tomato and affects the partitioning of Na + between plant organs. Plant, Cell Environ 32(7):904–916. 10.1111/j.1365-3040.2009.01971.x 19302170 10.1111/j.1365-3040.2009.01971.x

[CR72] Othman Y, Al-Karaki G, Al-Tawaha AR, Al-Horani A (2006) Variation in germination and ion uptake in barley genotypes under salinity conditions. World J Agric Sci 2(1):11–15

[CR73] Pfaffl MW (2001) A new mathematical model for relative quantification in real-time RT–PCR. Nucleic Acids Res 29(9):e45–e45. 10.1093/nar/29.9.e45 11328886 10.1093/nar/29.9.e45 PMC55695

[CR74] Prieto P, Pineda M, Aguilar M (1999) Spectrophotometric quantitation of antioxidant capacity through the formation of a phosphomolybdenum complex: specific application to the determination of vitamin E. Anal Biochem 269(2):337–341. 10.1006/abio.1999.4019 10222007 10.1006/abio.1999.4019

[CR75] Qiu L, Wu D, Ali S, Cai S, Dai F, Jin X, Wu F, Zhang G (2011) Evaluation of salinity tolerance and analysis of allelic function of HvHKT1 and HvHKT2 in Tibetan wild barley. Theor Appl Genet 122(4):695–703. 10.1007/s00122-010-1479-2 20981400 10.1007/s00122-010-1479-2

[CR76] Rady MM, Talaat NB, Abdelhamid MT, Shawky BT, Desoky E-SM (2019) Maize (Zea mays L.) grains extract mitigates the deleterious effects of salt stress on common bean (Phaseolus vulgaris L.) growth and physiology. J Hortic Sci Biotechnol 94(6):777–789. 10.1080/14620316.2019.1626773

[CR77] Rajabi Dehnavi A, Zahedi M, Piernik A (2024) Understanding salinity stress responses in sorghum: exploring genotype variability and salt tolerance mechanisms. Front Plant Sci 14:1296286. 10.3389/fpls.2023.1296286 38269142 10.3389/fpls.2023.1296286 PMC10806974

[CR78] Rathert G (1984) Sucrose and starch content of plant parts as a possible indicator for salt tolerance. Funct Plant Biol 11(6):491–495. 10.18006/2023.11(2).297.305

[CR80] Sabagh AEL, Hossain A, Islam MS, Barutcular C, Hussain S, Hasanuzzaman M, Akram T, Mubeen M, Nasim W, Fahad S (2019) Drought and salinity stresses in barley: consequences and mitigation strategies. Aust J Crop Sci 13(6):810–820

[CR81] Sadasivam S, Manickam A (1992) Biochemical methods for agricultural sciences. Wiley eastern limited

[CR82] Sahbeni G, Ngabire M, Musyimi PK, Székely B (2023) Challenges and opportunities in remote sensing for soil salinization mapping and monitoring: a review. Remote Sens 15(10):2540. 10.3390/rs15102540

[CR83] Schulte D, Close TJ, Graner A, Langridge P, Matsumoto T, Muehlbauer G, Sato K, Schulman AH, Waugh R, Wise RP (2009) The international barley sequencing consortium—at the threshold of efficient access to the barley genome. Plant Physiol 149(1):142–147. 10.1104/pp.108.128967 19126706 10.1104/pp.108.128967 PMC2613708

[CR84] Seleiman MF, Almutairi KF, Alotaibi M, Shami A, Alhammad BA, Battaglia ML (2020) Nano-fertilization as an emerging fertilization technique: why can modern agriculture benefit from its use? Plants Basel 10(1):2. 10.3390/plants10010002 33375026 10.3390/plants10010002 PMC7822031

[CR85] Sen A, Alikamanoglu S (2011) Effect of salt stress on growth parameters and antioxidant enzymes of different wheat (Triticum aestivum L.) varieties on in vitro tissue culture. Fresenius Environ Bull 20(2):489–495

[CR86] Shah T, Latif S, Saeed F, Ali I, Ullah S, Alsahli AA, Jan S, Ahmad P (2021) Seed priming with titanium dioxide nanoparticles enhances seed vigor, leaf water status, and antioxidant enzyme activities in maize (Zea mays L.) under salinity stress. J King Saud Univ-Sci 33(1):101207. 10.1016/j.jksus.2020.10.004

[CR87] Sharma S, Singh VK, Kumar A, Mallubhotla S (2019) Effect of nanoparticles on oxidative damage and antioxidant defense system in plants. Molecular Plant Abiotic: Stress Biology and Biotechnology. 10.1002/9781119463665.ch17

[CR88] Shen Q, Yu J, Fu L, Wu L, Dai F, Jiang L, Wu D, Zhang G (2018) Ionomic, metabolomic and proteomic analyses reveal molecular mechanisms of root adaption to salt stress in Tibetan wild barley. Plant Physiol Biochem 123:319–330. 10.1016/j.plaphy.2017.12.032 29289898 10.1016/j.plaphy.2017.12.032

[CR89] Shi H, Quintero FJ, Pardo JM, Zhu J-K (2002) The putative plasma membrane Na+/H+ antiporter SOS1 controls long-distance Na+ transport in plants. Plant Cell 14(2):465–477. 10.1105/tpc.010371 11884687 10.1105/tpc.010371 PMC152925

[CR90] Singh A, Rajput VD, Lalotra S, Agrawal S, Ghazaryan K, Singh J, Minkina T, Rajput P, Mandzhieva S, Alexiou A (2024) Zinc oxide nanoparticles influence on plant tolerance to salinity stress: insights into physiological, biochemical, and molecular responses. Environ Geochem Health 46(5):148 38578547 10.1007/s10653-024-01921-8

[CR91] Singh KM, Jha AB, Dubey RS, Sharma P (2025) Nanoparticle-mediated mitigation of salt stress-induced oxidative damage in plants: insights into signaling, gene expression, and antioxidant mechanisms. Environ Sci Nano 12(6):2983–3017

[CR92] Sonawane H, Arya S, Math S, Shelke D (2021) Myco-synthesized silver and titanium oxide nanoparticles as seed priming agents to promote seed germination and seedling growth of *Solanum lycopersicum*: a comparative study. Int Nano Lett 11(4):371–379. 10.1007/s40089-021-00346-w

[CR93] Srivastav A, Ganjewala D, Singhal RK, Rajput VD, Minkina T, Voloshina M, Srivastava S, Shrivastava M (2021) Effect of ZnO nanoparticles on growth and biochemical responses of wheat and maize. Plants Basel 10(12):2556. 10.3390/plants10122556 34961025 10.3390/plants10122556 PMC8708393

[CR94] Sun L, Wang Y, Wang R, Wang R, Zhang P, Ju Q, Xu J (2020) Physiological, transcriptomic, and metabolomic analyses reveal zinc oxide nanoparticles modulate plant growth in tomato. Environ Sci Nano 7(11):3587–3604

[CR95] Taha RS et al (2020) Exogenous potassium treatments elevate salt tolerance and performances of Glycine max L. By boosting antioxidant defense system under actual saline field conditions. Agronomy 10(11):1741

[CR96] Tang D, Chen M, Huang X, Zhang G, Zeng L, Zhang G, Wu S, Wang Y (2023) SRplot: a free online platform for data visualization and graphing. PLoS ONE 18(11):e0294236. 10.1371/journal.pone.0294236 37943830 10.1371/journal.pone.0294236 PMC10635526

[CR97] Thabet SG, Moursi YS, Sallam A, Karam MA, Alqudah AM (2021) Genetic associations uncover candidate SNP markers and genes associated with salt tolerance during seedling developmental phase in barley. Environ Exp Bot 188:104499. 10.1016/j.envexpbot.2021.104499

[CR98] Utz HF (2011) PLABSTAT A computer program for statistical analysis of plant breeding experiments Version 3A. Institute of Plant Breeding Seed Science and Population Genetics, University Hohenheim, Stuttgart

[CR99] Vats S (2018) Biotic and abiotic stress tolerance in plants. Biotic and abiotic stress tolerance in plants. Springer, pp 1–367

[CR100] Visioni A, Al-Abdallat A, Elenien JA, Verma RPS, Gyawali S, Baum M (2019) Genomics and molecular breeding for improving tolerance to abiotic stress in barley (Hordeum vulgare L.). Genomics Assisted Breeding of Crops for Abiotic Stress Tolerance, vol II. Springer, pp 49–68

[CR101] Wahid I, Kumari S, Ahmad R, Hussain SJ, Alamri S, Siddiqui MH, Khan MIR (2020) Silver nanoparticle regulates salt tolerance in wheat through changes in ABA concentration, ion homeostasis, and defense systems. Biomolecules 10(11):1506. 10.3390/biom10111506 33147820 10.3390/biom10111506 PMC7694077

[CR102] Widodo, Patterson JH, Newbigin ED, Tester M, Bacic A, Roessner U (2009) Metabolic responses to salt stress of barley (Hordeum vulgare L.) cultivars, Sahara and Clipper, which differ in salinity tolerance. J Exp Botany 60(14):4089–4103. 10.1093/jxb/erp243 19666960 10.1093/jxb/erp243 PMC2755029

[CR103] Xue D, Huang Y, Zhang X, Wei K, Westcott S, Li C, Chen M, Zhang G, Lance R (2009) Identification of QTLs associated with salinity tolerance at late growth stage in barley. Euphytica 169(2):187–196. 10.1007/s10681-009-9919-2

[CR104] Yang W, Rich PJ, Axtell JD, Wood KV, Bonham CC, Ejeta G, Mickelbart MV, Rhodes D (2003) Genotypic variation for glycinebetaine in sorghum. Crop Sci 43(1):162–169. 10.2135/cropsci2003.1620

[CR105] Yousefi Rad S, Soltanloo H, Ramezanpour SS, Zaynali Nezhad K (2019) The study of SOS genes expression in mutant barley root under salt stress. J Crop Breed 11(29):1–8

[CR106] Zhang J-L, Shi H (2013) Physiological and molecular mechanisms of plant salt tolerance. Photosynth Res 115(1):1–22. 10.1007/s11120-013-9813-6 23539361 10.1007/s11120-013-9813-6

[CR107] Zlobin IE (2021) Current understanding of plant zinc homeostasis regulation mechanisms. Plant Physiol Biochem 162:327–335. 10.1016/j.plaphy.2021.03.003 33714765 10.1016/j.plaphy.2021.03.003

[CR108] Zulfiqar F, Ashraf M (2021) Nanoparticles potentially mediate salt stress tolerance in plants. Plant Physiol Biochem 160:257–268. 10.1016/j.plaphy.2021.01.028 33529801 10.1016/j.plaphy.2021.01.028

