# Supplementary material for: Nano-priming modulates antioxidant enzymes and NHX/SOS-mediated ion homeostasis to improve salinity tolerance in barley genotypes
Source: Plant Cell Rep. 2026 Apr 13;45(5):122. doi: 10.1007/s00299-026-03798-6 (PMC13076421; doi:10.1007/s00299-026-03798-6)
Supplement: Supplementary file 1 — Supplementary file1 (PPTX 1259 KB) Fig. S1 Histogram of the difference in the biochemical traits under control and salinity stress between the most tolerant genotypes (A) and sensitive (B). Where, Protein (Pro), Carbohydrates (C), Sodium (Na), Potassium (K), Potassium/sodium ratio (K/Na), Sodium/potassium ratio (Na/K), Phosphorus (P), Peroxidase (POD), Glutathione-S-Transferase (GST), Catalase (CAT), and Total antioxidant capacity (TAC)Fig. S2 The fold change of each genotype under salinity compared to control for all traits (Protein content, Carbohydrate content, Proline content, Sodium ion, Potassium ion, K/Na ratio, Na/K ratio, Phosphorus content, Peroxidase activity, Glutathione_S_Transferase activity, Catalase activity, and Total Antioxidant Capacity) under all treatments (unprimed, hydro-priming, and nano-priming conditions)Fig. S3 Principal component analysis for 10 contrasting barley genotypes under control and salinity for all treatments; a) under unprimed conditions (UP), b) under hydro-priming conditions (H), and c) under nano-priming conditions (N). Where, Pro, C, Pr, Na, K, K/Na, Na/K, P, POD, GST, CAT, and TAC refer to protein, carbohydrate, proline, sodium, potassium, potassium/sodium ratio, sodium/ potassium ratio, phosphorus, peroxidase, glutathione-s-transferase, catalase, and total antioxidant capacityFig. S4 Clustering heatmap for 10 contrasting barley genotypes under control and salinity for all treatments a) control under unprimed conditions (C_UP), b) salinity under unprimed conditions (S_UP), c) control under hydro-priming conditions (C_H), d) salinity under hydro-priming conditions (S_H), e) control under nano-priming conditions (C_N), and f) salinity under nano-priming conditions (S_N). Where, Pro, C, Pr, Na, K, K/Na, Na/K, P, POD, GST, CAT, and TAC refer to protein, carbohydrate, proline, sodium, potassium, potassium/sodium ratio, sodium/ potassium ratio, phosphorus, peroxidase, glutathione-s-transferase, catalase, and total antioxidant ca [file 299_2026_3798_MOESM1_ESM.pptx]

## Slide 1
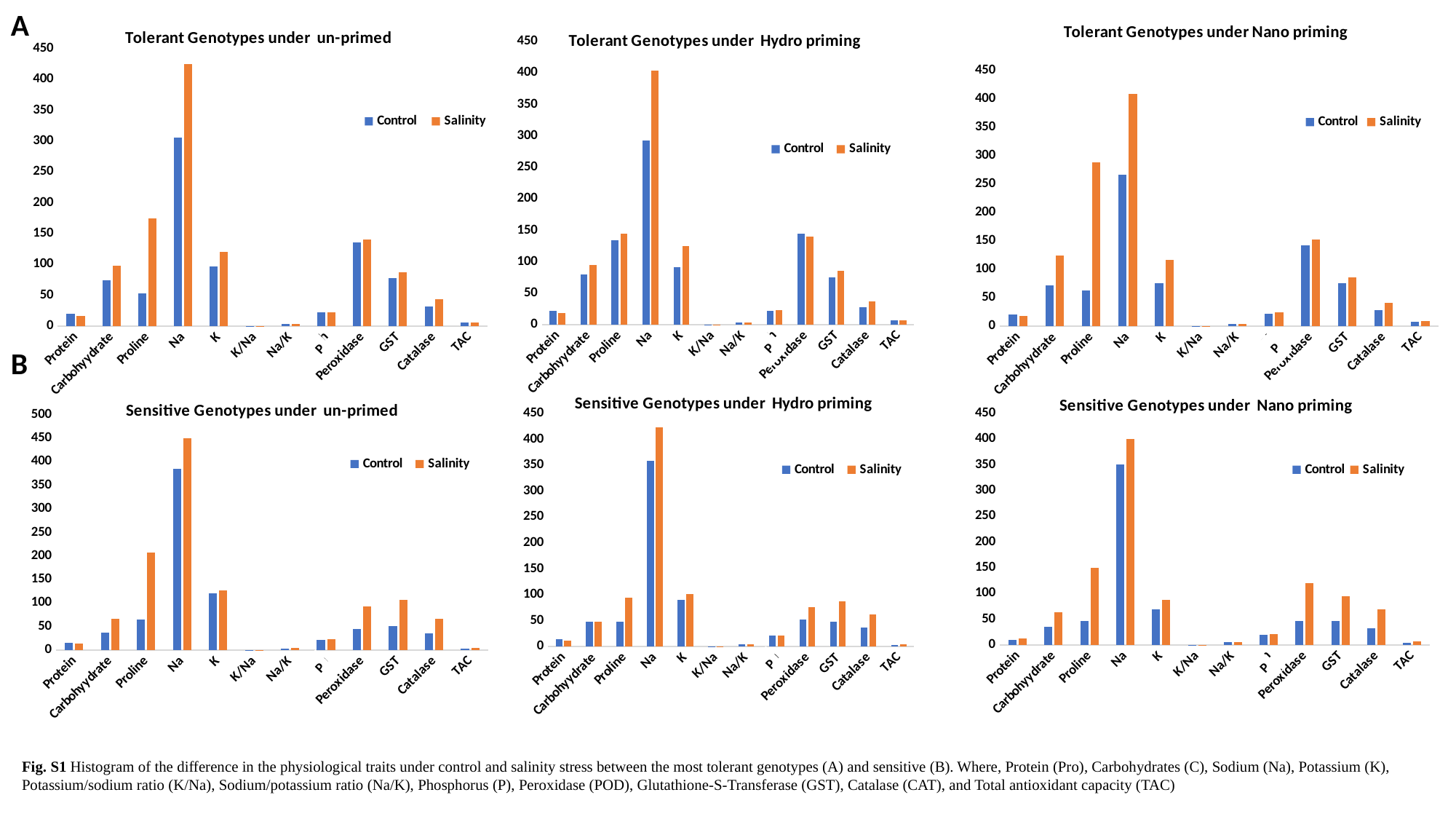

A
### Chart: Tolerant Genotypes under Nano priming
| Category | Control | Salinity |
|---|---|---|
| Protein | 21.240000000000002 | 18.686666666666667 |
| Carbohyydrate | 71.64 | 123.84666666666666 |
| Proline | 63.33333333333333 | 288.46666666666664 |
| Na | 266.2 | 408.53333333333336 |
| K | 76.16666666666667 | 116.66666666666667 |
| K/Na | 0.285557256661697 | 0.27190561194126533 |
| Na/K | 3.539758140499713 | 4.35467403423713 |
| Ph | 21.513333333333335 | 24.2 |
| Peroxidase | 142.16666666666669 | 152.86666666666665 |
| GST | 75.73333333333333 | 86.07333333333334 |
| Catalase | 28.073333333333334 | 41.38666666666666 |
| TAC | 7.775333333333333 | 8.725999999999999 |P
### Chart: Tolerant Genotypes under Hydro priming
| Category | Control | Salinity |
|---|---|---|
| Protein | 22.413333333333334 | 17.98 |
| Carbohyydrate | 80.40666666666667 | 95.38 |
| Proline | 133.96666666666664 | 144.3 |
| Na | 292.5333333333333 | 404.2 |
| K | 91.53333333333333 | 124.83333333333333 |
| K/Na | 0.31326569284819467 | 0.3043224004238051 |
| Na/K | 3.61511674923603 | 3.881566410989086 |
| Ph | 21.700000000000003 | 23.366666666666667 |
| Peroxidase | 144.73333333333335 | 140.6 |
| GST | 74.86666666666667 | 86.11333333333332 |
| Catalase | 27.54 | 37.53333333333334 |
| TAC | 7.078 | 6.968666666666667 |P
### Chart: Tolerant Genotypes under un-primed
| Category | Control | Salinity |
|---|---|---|
| Protein | 20.426666666666666 | 15.913333333333332 |
| Carbohyydrate | 74.52666666666667 | 98.31333333333333 |
| Proline | 52.66666666666667 | 174.86666666666665 |
| Na | 306.0 | 424.2 |
| K | 96.93333333333332 | 119.96666666666665 |
| K/Na | 0.3108681747862462 | 0.27869831944822765 |
| Na/K | 3.386103817118799 | 4.108976675817158 |
| Ph | 22.486666666666668 | 22.326666666666668 |
| Peroxidase | 135.2 | 140.73333333333335 |
| GST | 77.54666666666667 | 86.90666666666667 |
| Catalase | 31.55333333333333 | 43.339999999999996 |
| TAC | 5.691999999999999 | 6.017333333333334 |P
### Chart: Sensitive Genotypes under un-primed
| Category | Control | Salinity |
|---|---|---|
| Protein | 14.64 | 14.393333333333334 |
| Carbohyydrate | 36.77333333333333 | 66.29333333333334 |
| Proline | 64.66666666666666 | 206.66666666666666 |
| Na | 385.33333333333337 | 450.33333333333337 |
| K | 120.6 | 126.86666666666667 |
| K/Na | 0.3049964244783935 | 0.28873147081167305 |
| Na/K | 3.4861588504651357 | 3.91782536661252 |
| Ph | 21.233333333333334 | 22.593333333333334 |
| Peroxidase | 44.980000000000004 | 93.32 |
| GST | 51.4 | 106.06666666666665 |
| Catalase | 36.06 | 66.55333333333334 |
| TAC | 3.203333333333333 | 4.527333333333333 |P
### Chart: Sensitive Genotypes under Hydro priming
| Category | Control | Salinity |
|---|---|---|
| Protein | 14.5 | 11.920000000000002 |
| Carbohyydrate | 47.37333333333333 | 48.59333333333333 |
| Proline | 48.739999999999995 | 94.91333333333333 |
| Na | 358.1333333333333 | 422.6666666666667 |
| K | 90.3 | 101.7 |
| K/Na | 0.2430637840871157 | 0.24196716550776368 |
| Na/K | 4.286473394530958 | 4.329987836114613 |
| Ph | 20.659999999999997 | 21.773333333333333 |
| Peroxidase | 52.84666666666667 | 76.28 |
| GST | 47.666666666666664 | 87.66666666666667 |
| Catalase | 36.22 | 61.46666666666666 |
| TAC | 3.4206666666666665 | 4.87 |P
### Chart: Sensitive Genotypes under Nano priming
| Category | Control | Salinity |
|---|---|---|
| Protein | 10.046666666666667 | 13.046666666666667 |
| Carbohyydrate | 35.14666666666666 | 64.03999999999999 |
| Proline | 46.6 | 149.26666666666668 |
| Na | 350.53333333333336 | 400.7333333333334 |
| K | 69.4 | 86.83333333333334 |
| K/Na | 0.19800432560288836 | 0.2247164454373726 |
| Na/K | 5.518074814386961 | 4.759742176372173 |
| Ph | 19.639999999999997 | 21.606666666666666 |
| Peroxidase | 46.13333333333334 | 119.39333333333335 |
| GST | 46.66666666666667 | 94.43333333333334 |
| Catalase | 32.35333333333333 | 69.06 |
| TAC | 3.4306666666666663 | 6.465999999999999 |P
Fig. S1 Histogram of the difference in the physiological traits under control and salinity stress between the most tolerant genotypes (A) and sensitive (B). Where, Protein (Pro), Carbohydrates (C), Sodium (Na), Potassium (K), Potassium/sodium ratio (K/Na), Sodium/potassium ratio (Na/K), Phosphorus (P), Peroxidase (POD), Glutathione-S-Transferase (GST), Catalase (CAT), and Total antioxidant capacity (TAC)
B

## Slide 2
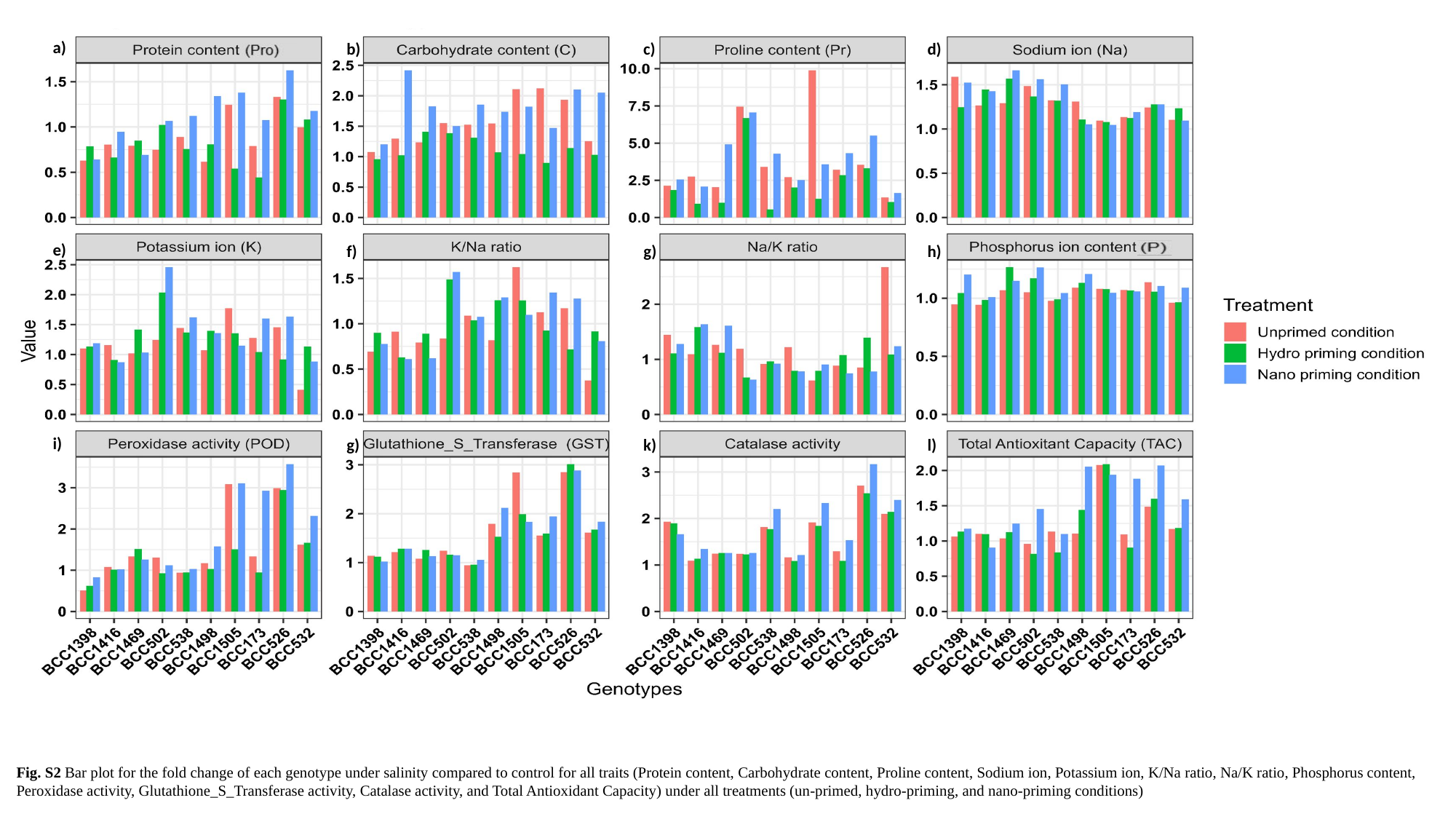

a)
b)
c)
d)
e)
g)
f)
h)
i)
k)
g)
l)
Fig. S2 Bar plot for the fold change of each genotype under salinity compared to control for all traits (Protein content, Carbohydrate content, Proline content, Sodium ion, Potassium ion, K/Na ratio, Na/K ratio, Phosphorus content, Peroxidase activity, Glutathione_S_Transferase activity, Catalase activity, and Total Antioxidant Capacity) under all treatments (un-primed, hydro-priming, and nano-priming conditions)
d)
a)
b)
c)
e)
f)
g)
h)
i)
j)
l)
k)

## Slide 3
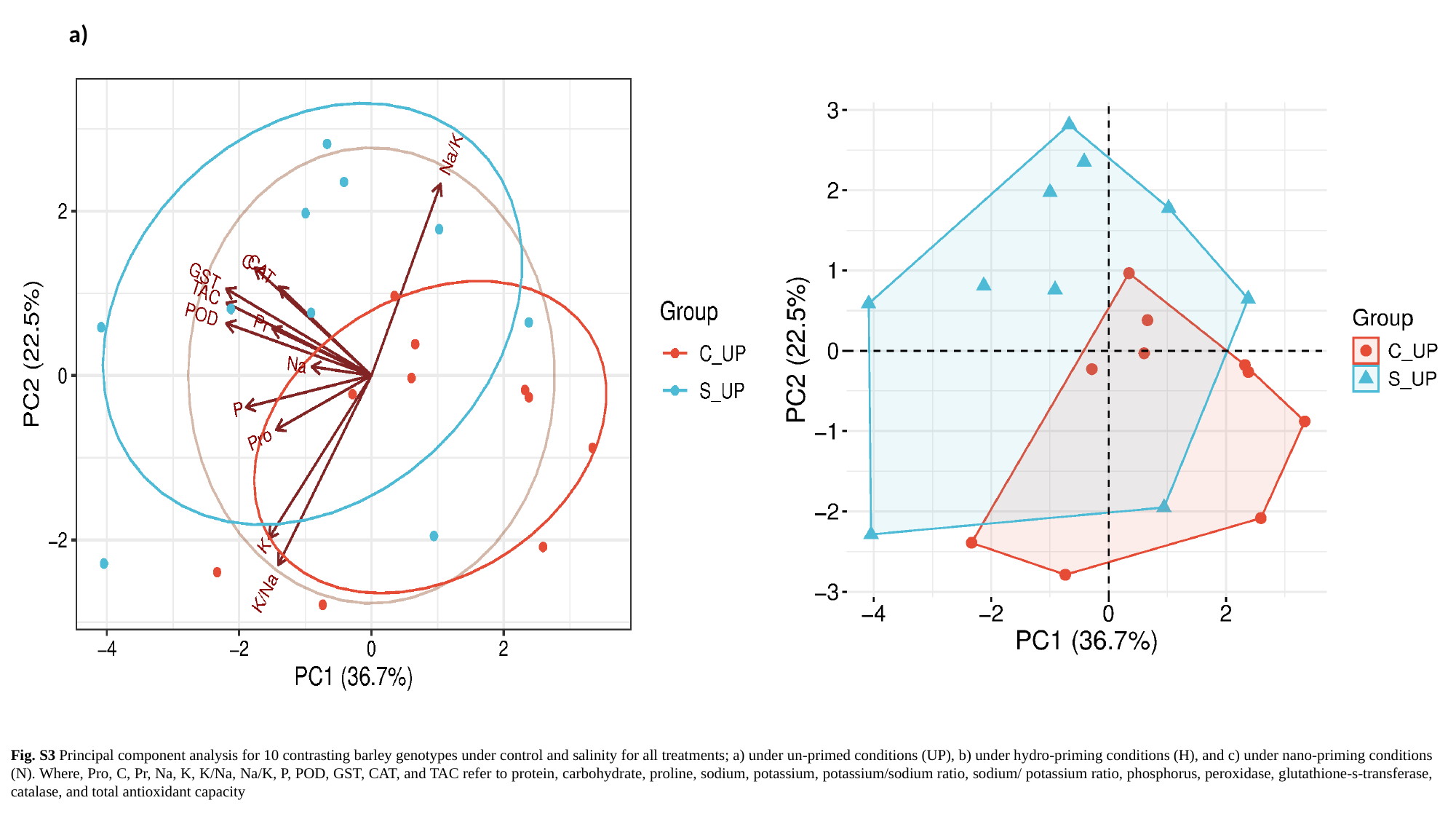

a)
Fig. S3 Principal component analysis for 10 contrasting barley genotypes under control and salinity for all treatments; a) under un-primed conditions (UP), b) under hydro-priming conditions (H), and c) under nano-priming conditions (N). Where, Pro, C, Pr, Na, K, K/Na, Na/K, P, POD, GST, CAT, and TAC refer to protein, carbohydrate, proline, sodium, potassium, potassium/sodium ratio, sodium/ potassium ratio, phosphorus, peroxidase, glutathione-s-transferase, catalase, and total antioxidant capacity

## Slide 4
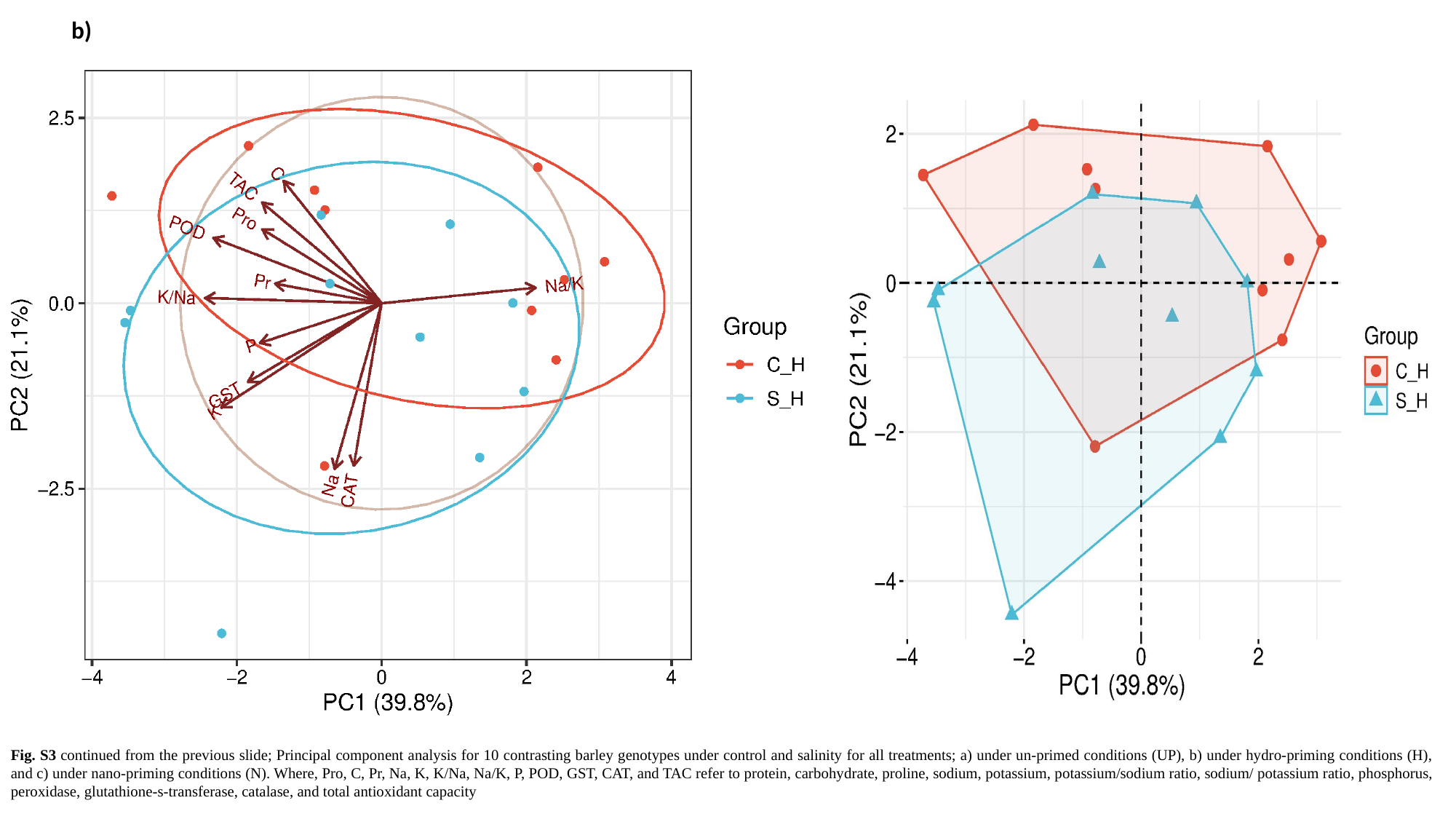

b)
Fig. S3 continued from the previous slide; Principal component analysis for 10 contrasting barley genotypes under control and salinity for all treatments; a) under un-primed conditions (UP), b) under hydro-priming conditions (H), and c) under nano-priming conditions (N). Where, Pro, C, Pr, Na, K, K/Na, Na/K, P, POD, GST, CAT, and TAC refer to protein, carbohydrate, proline, sodium, potassium, potassium/sodium ratio, sodium/ potassium ratio, phosphorus, peroxidase, glutathione-s-transferase, catalase, and total antioxidant capacity

## Slide 5
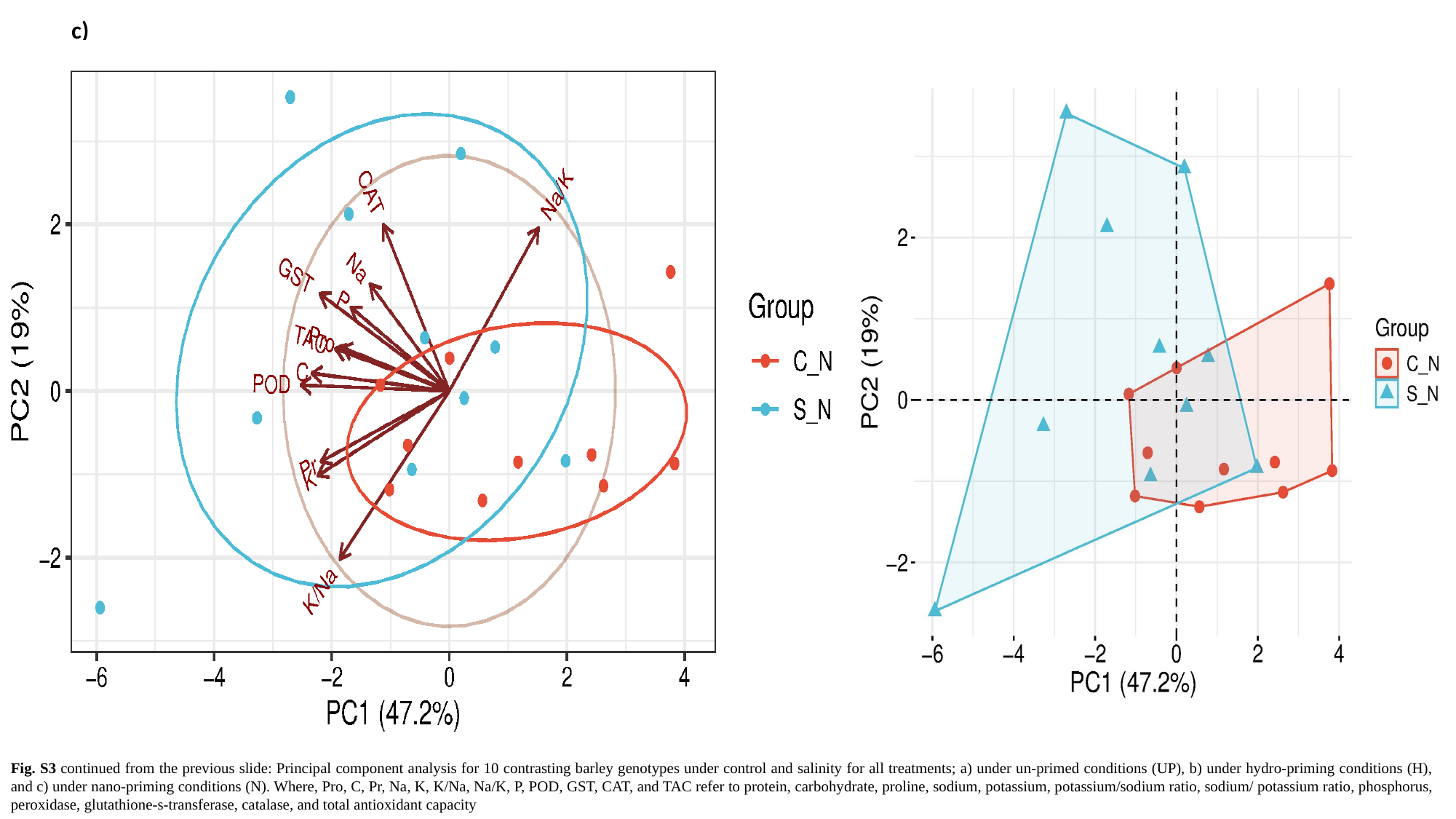

c)
Fig. S3 continued from the previous slide: Principal component analysis for 10 contrasting barley genotypes under control and salinity for all treatments; a) under un-primed conditions (UP), b) under hydro-priming conditions (H), and c) under nano-priming conditions (N). Where, Pro, C, Pr, Na, K, K/Na, Na/K, P, POD, GST, CAT, and TAC refer to protein, carbohydrate, proline, sodium, potassium, potassium/sodium ratio, sodium/ potassium ratio, phosphorus, peroxidase, glutathione-s-transferase, catalase, and total antioxidant capacity

## Slide 6
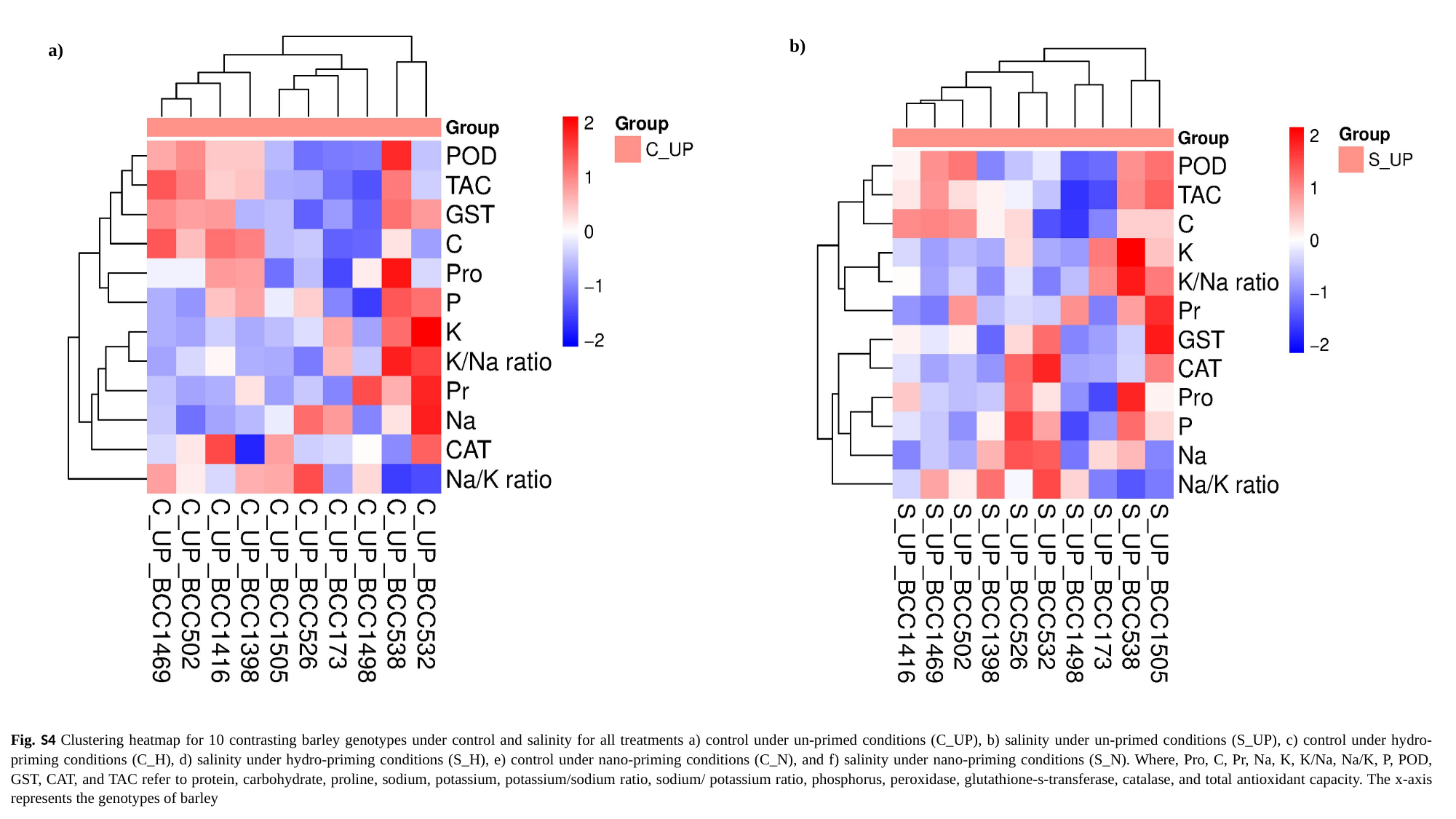

b)
a)
Fig. S4 Clustering heatmap for 10 contrasting barley genotypes under control and salinity for all treatments a) control under un-primed conditions (C_UP), b) salinity under un-primed conditions (S_UP), c) control under hydro-priming conditions (C_H), d) salinity under hydro-priming conditions (S_H), e) control under nano-priming conditions (C_N), and f) salinity under nano-priming conditions (S_N). Where, Pro, C, Pr, Na, K, K/Na, Na/K, P, POD, GST, CAT, and TAC refer to protein, carbohydrate, proline, sodium, potassium, potassium/sodium ratio, sodium/ potassium ratio, phosphorus, peroxidase, glutathione-s-transferase, catalase, and total antioxidant capacity. The x-axis represents the genotypes of barley

## Slide 7
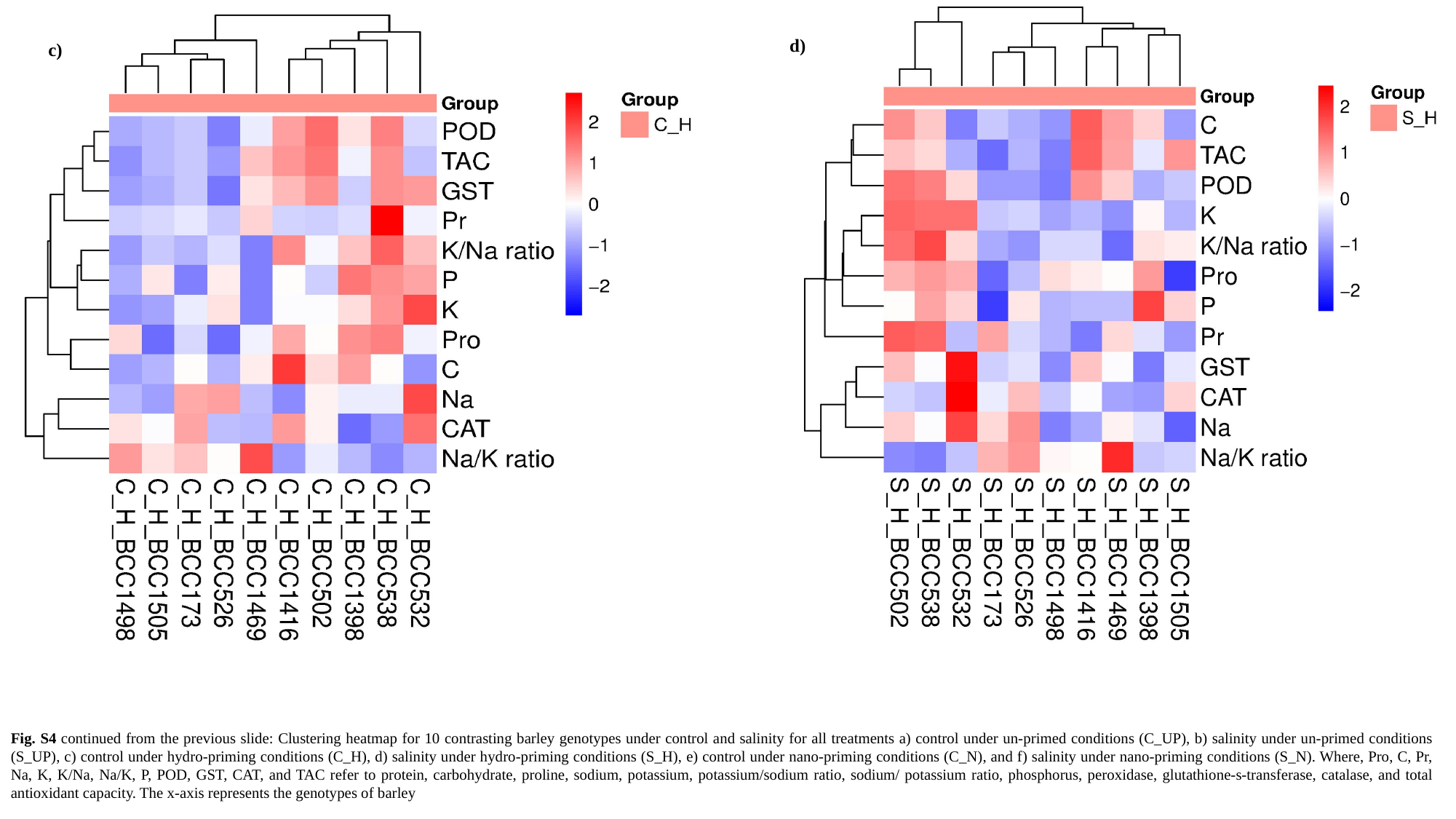

d)
c)
Fig. S4 continued from the previous slide: Clustering heatmap for 10 contrasting barley genotypes under control and salinity for all treatments a) control under un-primed conditions (C_UP), b) salinity under un-primed conditions (S_UP), c) control under hydro-priming conditions (C_H), d) salinity under hydro-priming conditions (S_H), e) control under nano-priming conditions (C_N), and f) salinity under nano-priming conditions (S_N). Where, Pro, C, Pr, Na, K, K/Na, Na/K, P, POD, GST, CAT, and TAC refer to protein, carbohydrate, proline, sodium, potassium, potassium/sodium ratio, sodium/ potassium ratio, phosphorus, peroxidase, glutathione-s-transferase, catalase, and total antioxidant capacity. The x-axis represents the genotypes of barley

## Slide 8
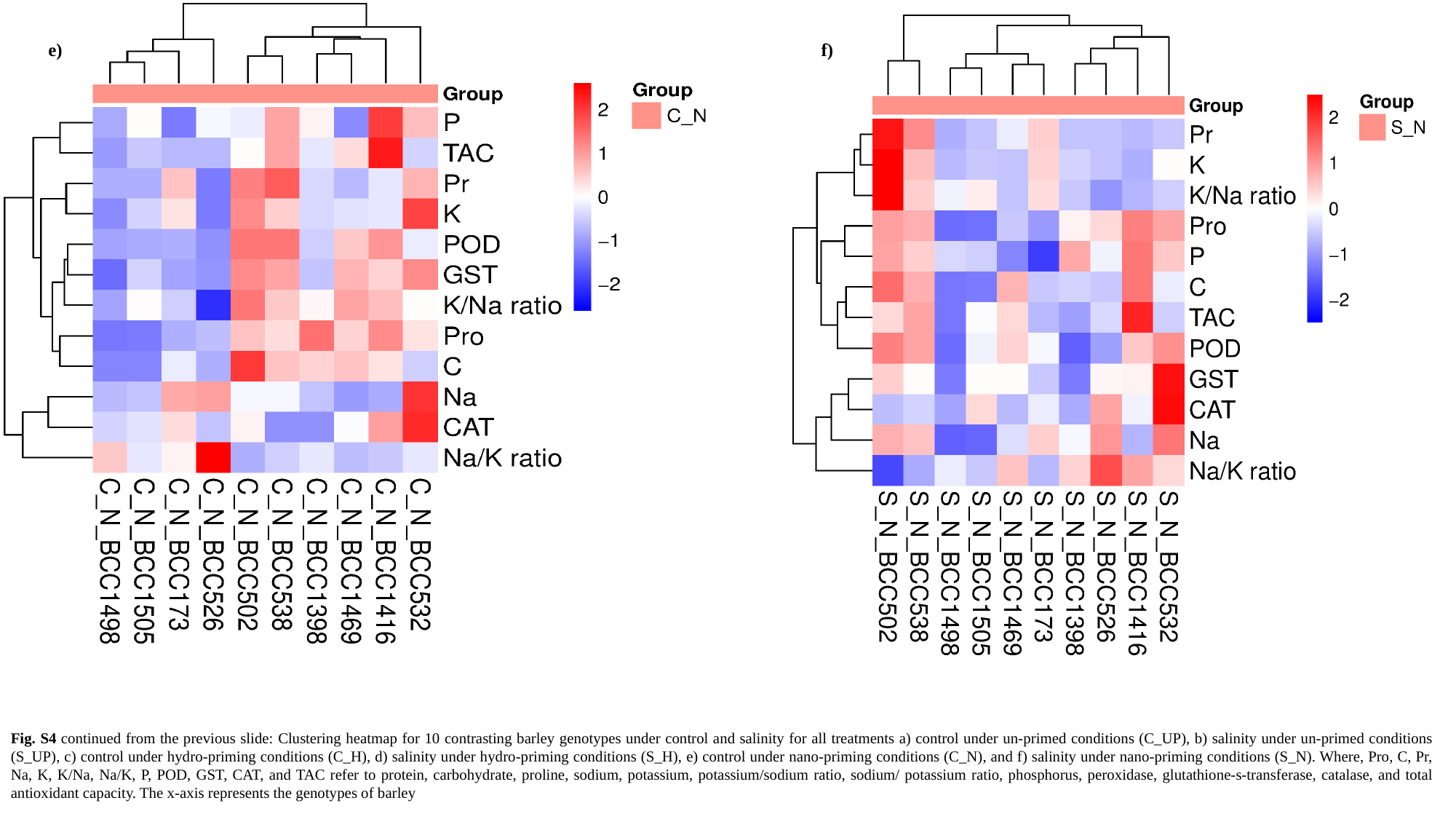

f)
e)
Fig. S4 continued from the previous slide: Clustering heatmap for 10 contrasting barley genotypes under control and salinity for all treatments a) control under un-primed conditions (C_UP), b) salinity under un-primed conditions (S_UP), c) control under hydro-priming conditions (C_H), d) salinity under hydro-priming conditions (S_H), e) control under nano-priming conditions (C_N), and f) salinity under nano-priming conditions (S_N). Where, Pro, C, Pr, Na, K, K/Na, Na/K, P, POD, GST, CAT, and TAC refer to protein, carbohydrate, proline, sodium, potassium, potassium/sodium ratio, sodium/ potassium ratio, phosphorus, peroxidase, glutathione-s-transferase, catalase, and total antioxidant capacity. The x-axis represents the genotypes of barley

## Slide 9
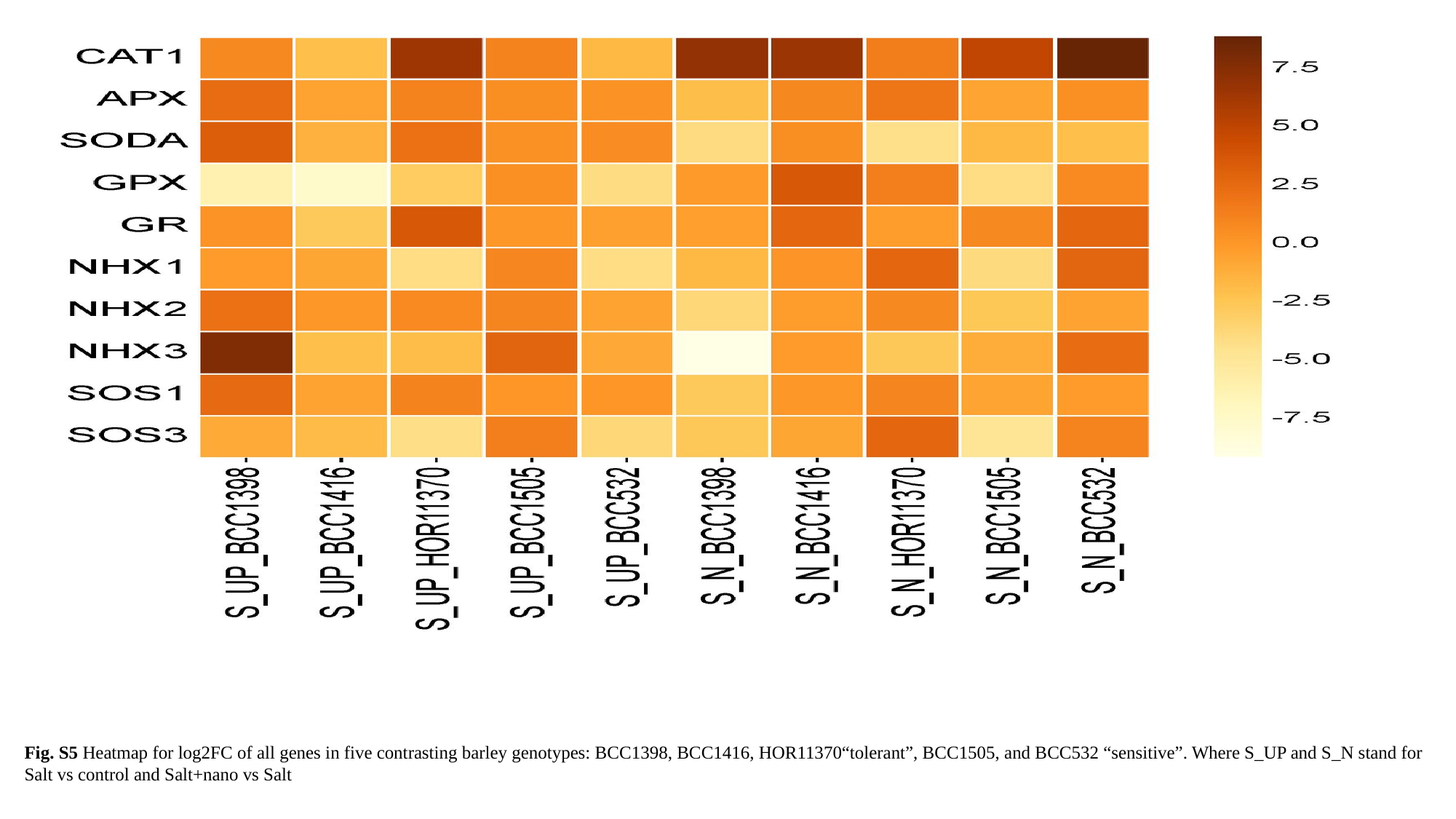

Fig. S5 Heatmap for log2FC of all genes in five contrasting barley genotypes: BCC1398, BCC1416, HOR11370“tolerant”, BCC1505, and BCC532 “sensitive”. Where S_UP and S_N stand for Salt vs control and Salt+nano vs Salt
